# The cell non-autonomous function of ATG-18 is essential for neuroendocrine regulation of *Caenorhabditis elegans* lifespan

**DOI:** 10.1371/journal.pgen.1006764

**Published:** 2017-05-30

**Authors:** Justin Minnerly, Jiuli Zhang, Thomas Parker, Tiffany Kaul, Kailiang Jia

**Affiliations:** Department of Biological Sciences, Florida Atlantic University, Jupiter, FL, United States of America; Princeton, UNITED STATES

## Abstract

Dietary restriction (DR) and reduced insulin growth factor (IGF) signaling extend lifespan in *Caenorhabditis elegans* and other eukaryotic organisms. Autophagy, an evolutionarily conserved lysosomal degradation pathway, has emerged as a central pathway regulated by various longevity signals including DR and IGF signaling in promoting longevity in a variety of eukaryotic organisms. However, the mechanism remains unclear. Here we show that the autophagy protein ATG-18 acts cell non-autonomously in neuronal and intestinal tissues to maintain *C*. *elegans* wildtype lifespan and to respond to DR and IGF-mediated longevity signaling. Moreover, ATG-18 activity in chemosensory neurons that are involved in food detection sufficiently mediates the effect of these longevity pathways. Additionally, ATG-18-mediated cell non-autonomous signaling depends on the release of neurotransmitters and neuropeptides. Interestingly, our data suggest that neuronal and intestinal ATG-18 acts in parallel and converges on unidentified neurons that secrete neuropeptides to regulate *C*. *elegans* lifespan through the transcription factor DAF-16/FOXO in response to reduced IGF signaling.

## Author summary

Restriction of dietary food without malnutrition robustly extends lifespan in more than twenty species. It was also reported that fruit flies (*Drosophila melanogaster*) deficient in olfactory function live longer and that the longevity induced by food restriction is partially due to decreased olfaction. These observations suggest food assimilation through the gastrointestinal tract and food smell detected by olfactory neurons influence lifespan. The insulin growth factor signaling pathway is regulated by nutrient levels and has been shown to mediate the lifespan extension conferred by food restriction and defective gustatory neurons in the nematode *Caenorhabditis elegans*. However, the mechanism remains unclear. Autophagy is a lysosomal degradation pathway and is sensitive to nutrient availability. We found autophagy activity in the intestine and food sensory neurons acts in parallel to mediate food restriction and insulin signaling regulated lifespan extension in *Caenorhabditis elegans*. Moreover, intestinal and neuronal autophagy converges on unidentified neurons to control the secretion of neuropeptides that regulate lifespan. These data suggest autophagy is an essential component in a neuroendocrine pathway that coordinates how environmental food cues detected by sensory neurons and food nutrients assimilated by the intestine influence lifespan. These findings may contribute to understanding the aging process in mammals.

## Introduction

Limitation of food without causing malnutrition (DR: dietary restriction) extends lifespan in a wide range of species [1]. In *C*. *elegans*, multiple signaling transduction pathways are involved in response to DR, including the insulin-like growth factor pathway (IGF) [1]. The *C*. *elegans daf-2* gene encodes an insulin receptor-like tyrosine kinase [2]. DAF-16, a FOXO (forkhead box O) transcription factor, is a major target of DAF-2 signaling and its activity is required for the longevity of *daf-2* mutants [3, 4]. When DAF-2 signaling is inhibited, DAF-16 enters the nucleus and turns on the transcription of many longevity genes to increase lifespan [5]. Under certain DR conditions DAF-2 signaling mediates the response to DR [6]. However, the IGF and DR pathways can be separated as DAF-2 is not required for lifespan extension of *C*. *elegans* under all DR conditions [7, 8].

DAF-2 signaling has also been linked to lifespan extension resulted from defective sensory neurons [9]. *C*. *elegans* use amphid sensory neurons to detect environmental cues. The worm has one pair of thermosensory neurons and eleven pairs of bilaterally symmetric chemosensory neurons. The chemosensory neurons can detect a variety of olfactory and gustatory signals through their sensory cilia that penetrate the cuticle and are exposed to the external environment [10, 11]. These neurons allow worms to process and transmit environmental cues to neuronal or nonneuronal endocrine cells that can modulate hormonal signaling to adjust physiology to maintain homeostasis. Interestingly, several studies have shown that chemosensory neurons regulate lifespan in *C*. *elegans*. Mutations that disrupt sensory cilia or laser ablation of their support cells, resulting in defective sensory perception, were able to increase lifespan [12]. Laser ablation of either ASI or ASG gustatory neurons or AWA or AWC olfactory neurons alone extends lifespan [13]. Moreover, the olfactory and gustatory neurons act in parallel to control lifespan [13]. Lifespan extension due to ablating gustatory neurons but not olfactory neurons is dependent on DAF-16 and ablation of gustatory neurons cannot further increase the lifespan of *daf-2* mutants, indicating gustatory neurons function through DAF-2 [13]. Similar to *C*. *elegans*, in the fruit fly *Drosophila melanogaster* loss of olfactory function increases lifespan. Furthermore, DR was less effective when olfactory mutant flies were exposed to food odors, suggesting DR longevity is partially due to decreased olfaction [14]. Despite these observations, the molecular mechanism by which environmental food cues and food nutrients coordinate to influence lifespan remains elusive.

Macroautophagy (hereafter autophagy) has emerged as a central pathway regulating aging in various model organisms including *C*. *elegans* and is essential for both DR and IGF signaling induced longevity [15–17]. Autophagy is highly sensitive to food availability and strongly induced by starvation. Upon induction, double-membrane vesicles referred to as autophagosomes form, engulf cellular components and then fuse with lysosomes allowing the sequestered contents to be degraded. The degraded products are recycled to synthesize new macromolecules and maintain cellular energy homeostasis [18, 19].

The autophagosome is derived from the elongation and closure of a membrane precursor called the phagophore [19]. ATG-18 is a member of the WIPI (WD repeat protein interacting with phosphoinositides) protein family that binds to phosphatidylinositol 3-phosphate to promote formation and elongation of the phagophore [20]. WIPI1 and WIPI2 are mammalian Atg18 homologs and both are protein components of autophagosomes [21, 22]. Autophagy is suppressed in the loss-of-function *C*. *elegans* null mutant *atg-18(gk378)*, as indicated by the accumulation of p62/sequestosome 1 (SQSTM1) (a cargo receptor for autophagic degradation of ubiquitinated protein aggregates) [23], the inefficient clearance of cell corpses [24, 25], exacerbation of polyglutamine protein toxicity [26] and decreased lifespan [27]. Interestingly, mammalian WIPI proteins are also involved in the pathogenesis of age-related human diseases such as cancer and neurodegenerative disorders [28], implying that the function of ATG-18 is evolutionarily conserved from *C*. *elegans* to humans, which highlights the importance of understanding the mechanisms by which ATG-18/WIPI proteins regulate the aging process.

Here we show that autophagy protein ATG-18 acts cell non-autonomously in the neurons and intestine to maintain *C*. *elegans* wildtype lifespan and respond to DR and DAF-2 longevity signals. Furthermore, ATG-18 activity in chemosensory neurons is sufficient to mediate the effect of IGF signaling and DR on lifespan. In addition, we found that neuronal and intestinal ATG-18 converges on unidentified neurons to control *C*. *elegans* lifespan through neuropeptide signals.

## Results

### Tissue-specific requirement of ATG-18 in maintenance of wildtype lifespan

**Fig 1:**
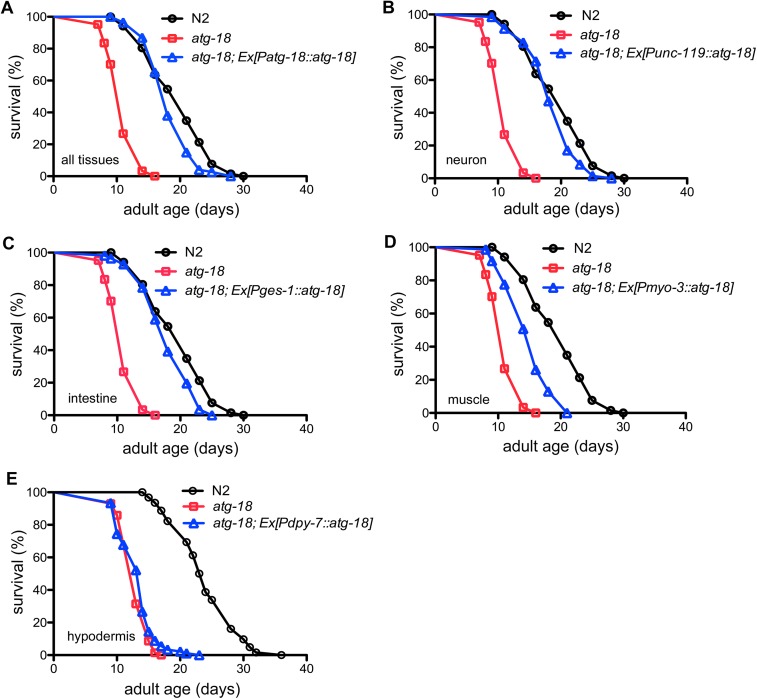
Effect of tissue-specific ATG-18 activity on the lifespan of *atg-18(gk378)* mutants. Natively-expressed ATG-18 (A) or expression of *atg-18* in neurons (B) and intestinal cells (C) restores the lifespan of *atg-18* mutants to wildtype levels. (D) Muscle ATG-18 modestly increases the lifespan of *atg-18* mutants. (E) Hypodermal ATG-18 has no effect on the lifespan of *atg-18* mutants.

We examined the tissue requirement of *atg-18* for regulating *C*. *elegans* wildtype lifespan. ATG-18 is expressed in almost every tissue including the body wall muscles, hypodermis, neurons and intestine [29]. As the *atg-18* mutant is short-lived [27], we used a complementation approach to express *atg-18* in different tissues to examine where autophagy is required for maintenance of a wildtype lifespan. We found the full-length genomic DNA of *atg-18* whose expression is driven by its native promoter (*Patg-18*::*atg-18*, Fig 1A, S1 Table), a pan-neuronal promoter (*Punc-119*::*atg-18*, Fig 1B) and an intestine-specific promoter (*Pges-1*::*atg-18*, Fig 1C) fully restores the lifespan of *atg-18* mutants to that of wildtype N2 worms. Expression of *atg-18* in body wall muscles (*Pmyo-3*::*atg-18*, Fig 1D) modestly rescues the short lifespan of *atg-18* mutants. However, hypodermal expression of *atg-18* (*Pdpy-7*::*atg-18*, Fig 1E) has no effect on wildtype lifespan. These results indicate that autophagy activity in neurons and intestinal cells is essential for maintenance of wildtype lifespan. We also expressed *atg-18* in the tissues where it is natively expressed in N2 animals and found that this overexpression of *atg-18* decreases the wildtype lifespan (S1 Fig, S2 Table).

### Tissue-specific requirement of ATG-18 in response to dietary restriction

**Fig 2:**
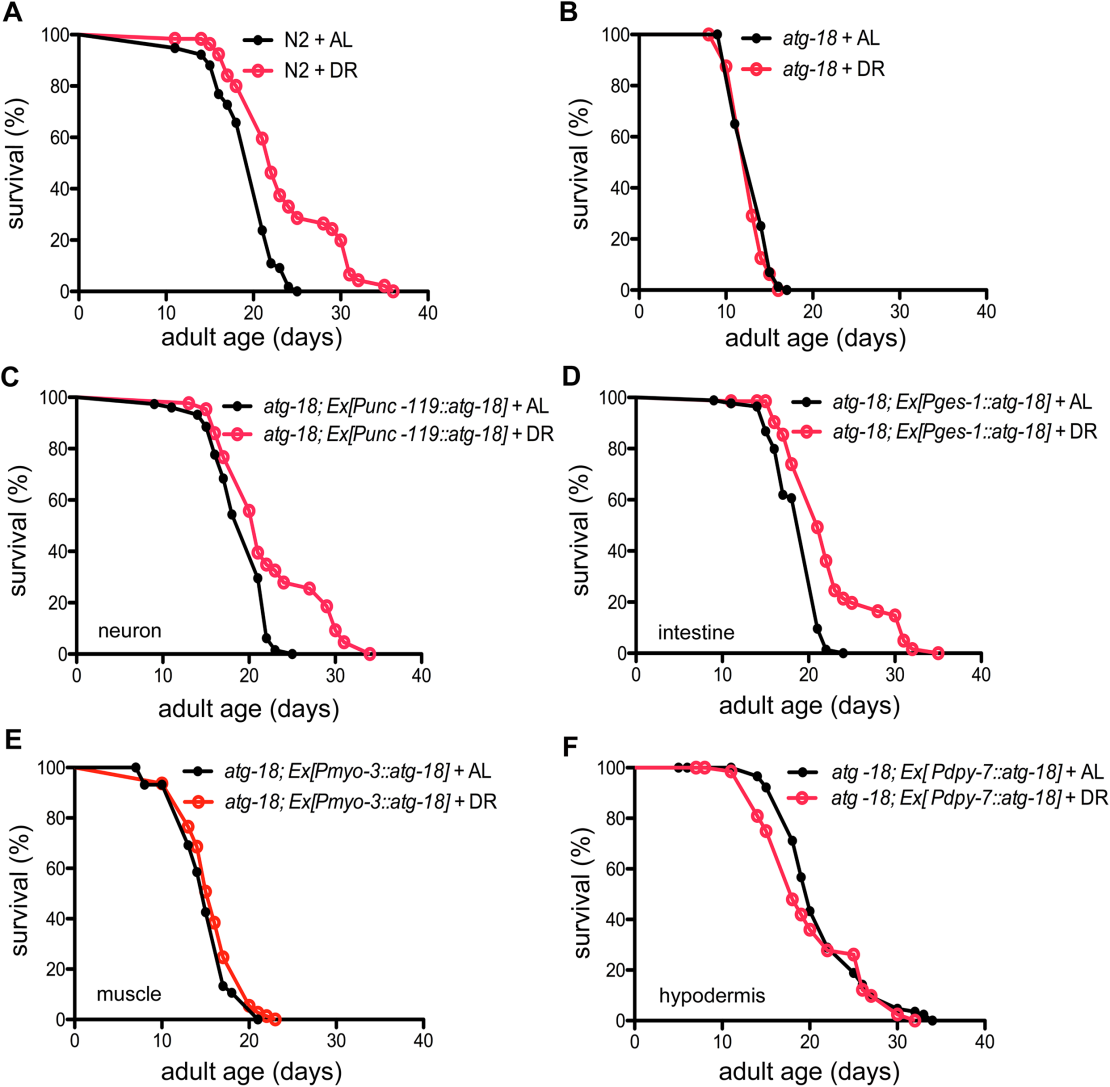
Tissue-specific requirement for *atg-18* in dietary restriction (DR) response compared to *ad libitum* feeding (AL). (A) Wildtype N2 animals live longer after DR treatment. (B) The *atg-18(gk378)* mutation blocks lifespan extension in response to DR. (C-D) Neuronal and intestinal ATG-18 restores the DR response in *atg-18* mutants. (E-F) Muscle and hypodermal ATG-18 has no influence on DR-mediated lifespan extension in *atg-18* mutants.

Autophagy genes are required for DR-mediated lifespan extension in *C*. *elegans* [30, 31]. We examined the tissue requirement of *atg-18* in response to solid DR (sDR: bacteria are serially diluted on plates) [32]. Similar to previous studies [32], the lifespan of N2 worms treated by sDR (bacteria concentration: 5X10^9^cells/mL) is significantly increased compared to control animals fed *ad libitum* (AL: bacteria concentration: 5X10^11^cells/mL) (Fig 2A, S3 Table) (P<0.0001, log-rank test). However, the *atg-18(gk378)* mutation completely abrogates the response to sDR (Fig 2B). We found the lifespan of *atg-18* worms with *atg-18* rescued in either neurons or intestinal cells is significantly increased after sDR treatment (Fig 2C and 2D). However, expression of *atg-18* in the body wall muscles or hypodermis fails to restore the DR longevity (Fig 2E and 2F). Thus, *atg-18* expression in neurons and intestinal cells plays a major role in the DR response.

### Tissue-specific requirement of ATG-18 for the longevity phenotype of *daf-2* mutants

**Fig 3:**
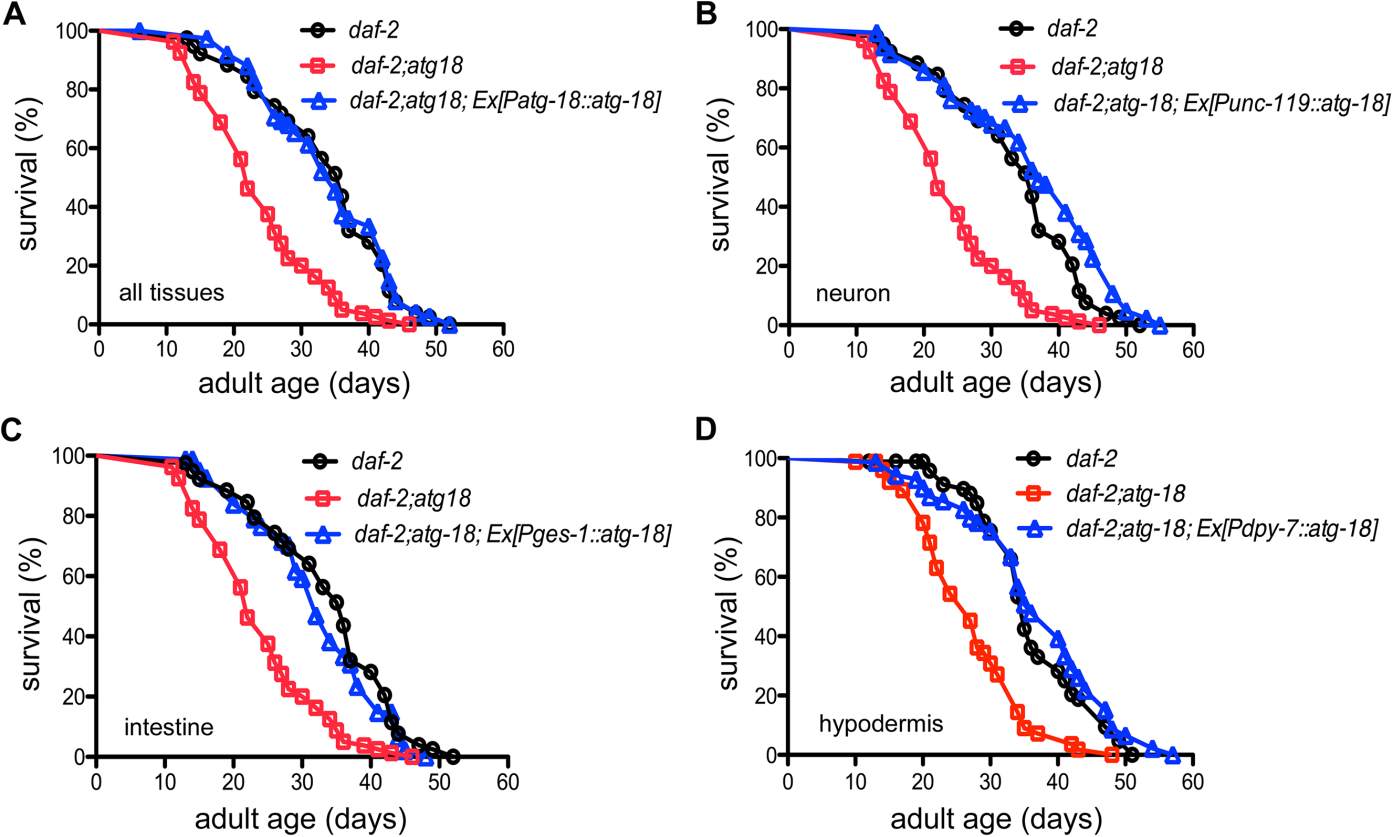
Effect of tissue-specific *atg-18* activity on the lifespan of *daf-2(e1370)* mutants. Natively-expressed ATG-18 (A) or expression of *atg-18* in neurons (B), intestinal cells (C) and hypodermis (D) restores the lifespan of *daf-2(e1370);atg-18(gk378)* mutants to *daf-2(e1370)* levels.

Similar to a previous report that ATG-18 is required for the longevity phenotype of *daf-2* mutants [33], the *atg-18(gk378)* mutation significantly suppresses the longevity phenotype of *daf-2(e1370)* worms (S2A Fig, S4 Table) (P<0.0001, log-rank test). When a natively expressed *atg-18* transgene was introduced into *daf-2(e1370);atg-18(gk378)* mutants, the longevity phenotype of *daf-2* mutants is completely restored (Fig 3A). Expression of *atg-18* in neurons or in intestinal cells also completely reverts the lifespan of *daf-2;atg-18* to that of *daf-2* mutants (Fig 3B and 3C). Expression of *atg-18* in body wall muscles modestly influences the lifespan of *daf-2;atg-18* mutants (S2B Fig, S4 Table). Interestingly, *daf-2;atg-18* mutants carrying hypodermal ATG-18 live as long as *daf-2* mutants (Fig 3D). To confirm that expression of *atg-18* in neurons alone can rescue the short lifespan of *daf-2;atg-18* mutants, we used the *rgef-1* promoter, another common pan-neuronal promoter [34], to express *atg-18* in the neurons of *daf-2;atg-18* mutants. We found that neuronal expression of *atg-18* under the control of the *rgef-1* promoter fully rescues the short lifespan of *daf-2;atg-18* mutants (S3 Fig, S5 Table).

### Influence of tissue-specific expression of *atg-18* on autophagy activity

**Fig 4:**
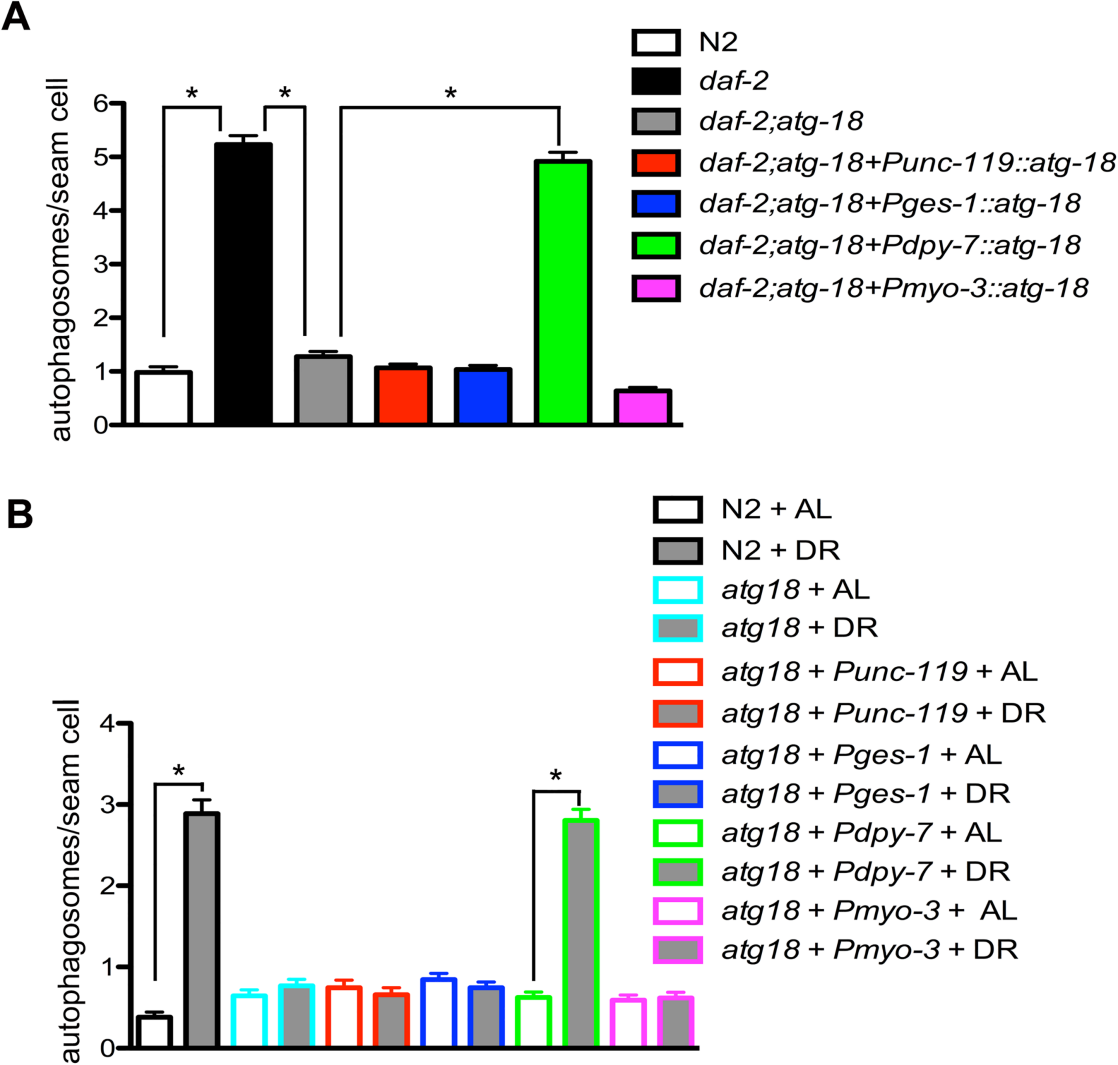
Influence of tissue-specific expression of *atg-18* on autophagy activity. Quantification of GFP::LGG-1 puncta in hypodermal seam cells is shown for the indicated genetic backgrounds and conditions. (A) Autophagy induction in *daf-2(e1370)* mutants is blocked by the *atg-18(gk378)* mutation. Expression of *atg-18* in the hypodermis but not in other tissues restores the autophagy induction in the hypodermis of *daf-2;atg-18* mutants. (B) DR induces autophagy induction in wildtype N2 worms but not in *atg-18* mutants. Hypodermal expression of *atg-18* restores autophagy induction in the hypodermis after DR treatment. However, expression of *atg-18* in neurons, intestinal cells or the muscles has no obvious influence on the autophagy activity in the hypodermis after DR treatment. Asterisks indicate statistically significant differences relative to controls (* P < 0.0001, t-test). The count of puncta per seam cell and statistical analysis are presented in S6 Table. Representative pictures of autophagosomes in seam cells are shown in S4 Fig.

We examined autophagy activity in the *daf-2* mutant background and in sDR-treated animals. The GFP::LGG-1 reporter was used to visualize autophagosomes that appear as fluorescence puncta in hypodermal seam cells [33, 35]. We found that the number of autophagosomes in the seam cells is greatly decreased in *daf-2;atg-18* mutants compared to the *daf-2* mutants (Fig 4A, S6 Table, S4B Fig, S4D Fig), indicating that autophagy activity is suppressed in *daf-2;atg-18* mutants. Moreover, expression of *atg-18* in neurons, intestinal cells and muscles fails to increase the number of autophagosomes in seam cells (Fig 4A, S6 Table, S4E Fig, S4F Fig, S4H Fig). By contrast hypodermal expression of *atg-18* restores the autophagy activity level in seam cells back to the *daf-2* mutant level (Fig 4A, S6 Table, S4G Fig).

Similar to observations about autophagy activity in *daf-2;atg-18* mutants, we found that sDR induces autophagy in wildtype N2 animals and this autophagy induction is suppressed in *atg-18* mutants as judged by the number of autophagosomes in hypodermal seam cells (Fig 4B, S6 Table, S4I Fig, S4J Fig, S4K Fig, S4L Fig). Although expression of *atg-18* in neurons, intestinal cells and muscles cannot restore autophagy induction in the seam cells of *atg-18* mutants upon sDR, autophagy is induced in the seam cells of *atg-18* mutants carrying hypodermally expressed *atg-18* (Fig 4B, S6 Table, S4M Fig, S4N Fig, S4O Fig, S4P Fig, S4Q Fig, S4R Fig, S4S Fig, S4T Fig). These data suggest that tissue-specific expression of *atg-18* in the *atg-18* mutant background can only restore autophagy in the tissues where the *atg-18* gene is expressed. Thus, under this condition ATG-18 in specific tissues should generate a cell non-autonomous signal to influence *C*. *elegans* lifespan instead of relying on autophagy induction in other tissues.

### ATG-18 in ADF, ADL, ASG or AWA chemosensory neurons sufficiently mediates the DAF-2 longevity signal

**Fig 5:**
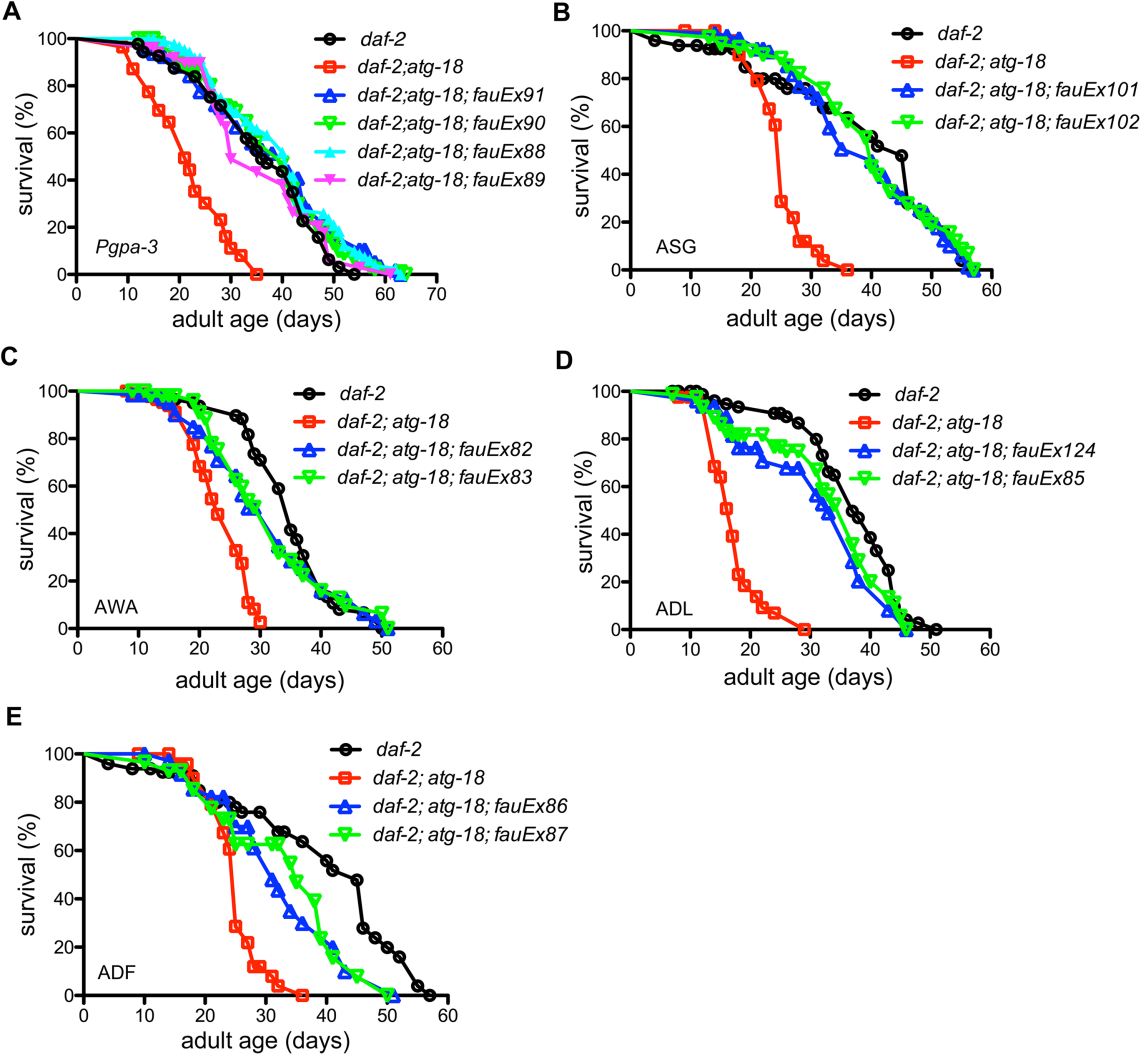
ATG-18 activity in chemosensory neurons is essential for IGF signaling to control lifespan. (A) ATG-18 in a subset of chemosensory neurons restores the lifespan of *daf-2(e1370);atg-18(gk378)* mutants to *daf-2(e1370)* levels. (B) *daf-2;atg-18* mutants carrying ATG-18 in ASG gustatory neurons have a similar lifespan to that of *daf-2* mutants. (C-E) ATG-18 in AWA (C) and ADL (D) olfactory neurons and in ADF serotonin neurons (E) significantly increase the lifespan *daf-2;atg-18* mutants (p<0.0001, log-rank test).

Expression of *atg-18* in ASH neurons and more than 20 other neurons (*Punc-42*::*atg-18*) does not increase the lifespan of *daf-2;atg-18* mutants (S5A Fig, S7 Table) (P = 0.1816 and 0.3174 for two independent transgenic lines, log-rank test). ATG-18 expressed in ASE, ASI, ASJ, ASK, AWB and AWC neurons (*Pdaf-11*::*atg-18*) also fails to extend *daf-2;atg-18* lifespan (S5B Fig) (P = 0.1865 and 0.5324 for two independent transgenic lines, log-rank test). By contrast, expression of *atg-18* in ADF, ADL, ASE, ASG, ASH, ASI, ASJ, ASK, AWA and AWC neurons (*Pgpa-3*::*atg-18*) fully restores lifespan of *daf-2;atg-18* to *daf-2(e1370)* levels (Fig 5A, S7 Table). We conclude that *atg-18* activity in ADF, ADL, ASG and AWA neurons is essential to mediate the effect of IGF signaling on lifespan. We expressed *atg-18* in ASG (*Podr-2*::*atg-18*), AWA (*Podr-10*::*atg-18*), ADL (*Psrh-220*::*atg-18*) and ADF (*Ptph-1*::*atg-18*) neurons individually and found it significantly increases *daf-2;atg-18* lifespan (Figs 5B–4E). Notably, ATG-18 in ASG gustatory neurons fully restores *daf-2(e1370)* longevity in *daf-2;atg-18* worms.

To confirm the expression of *atg-18* in individual chemosensory neurons, we inserted the *gfp* coding sequence upstream of *atg-18* genomic DNA in the same plasmids used for the individual neuronal rescue lifespan experiments. The expression pattern of these *gfp* reporter genes is consistent with previous publications. Two promoters, *odr-10* and *srh-220*, drive the expression of *atg-18* in single pairs of sensory neurons (S6B Fig, S6C Fig) [36]. The *Ptph-1*::*gfp*::*atg-18* reporter gene is expressed in serotonergic neurons including ADF, NSM and HSN neurons as reported previously (S6D Fig, S6E Fig) [37]. For the *odr-2* promoter, we used the same promoter reported by Chou et al. [38]. This *odr-2(2b)* promoter is active in AIZ, AIB, AVG, RIF, PVP, and RIV interneurons, SIAV motor neurons, and IL2 and ASG sensory neurons [38]. The expression the *Podr-2*::*gfp*::*atg-18* reporter gene was observed in six neurons posterior to the nerve ring. Based on their relative positions in the head, three neurons (ASG, AIB and AIZ) are labelled in S6A Fig. A similar expression pattern has been reported previously [38]. As expression of *atg-18* in the chemosensory neurons under control of the *gpa-3* promoter fully rescues the short lifespan of *daf-2;atg-18* mutants (Fig 5A), we conclude that it is the expression of *atg-18* in ASG neurons not the interneurons driven by the *odr-2* promoter that mediates the influence of *daf-2* signaling on the lifespan. Taken together, these data indicate that expression of *atg-18* in four chemosensory neurons is essential to mediate the effect of *daf-2* insulin-like signaling on *C*. *elegans* lifespan.

Next, we expressed *atg-18* in the chemosensory neurons of *atg-18* mutants under control of the *gpa-3* promoter. We found that the *Pgpa-3*::*atg-18* transgene only partially rescues the short lifespan of *atg-18* mutants (S7 Fig, S8 Table), which is in contrast to the observation that the *Pgpa-3*::*atg-18* transgene fully restores the lifespan of *daf-2;atg-18* mutants to the *daf-2* mutant level (Fig 5A). Thus, expression of *atg-18* in chemosensory neurons plays a more significant role in regulation of the *daf-2* mutant lifespan than that of *atg-18* mutants. Interestingly, *atg-18* mutants carrying an *atg-18* transgene in chemosensory neurons (*Pgpa-3*::*atg-18)* show increased lifespans after sDR (S8 Fig, S9 Table). Hence, ATG-18 in chemosensory neurons also influences the aging process regulated by DR in *C*. *elegans*.

### ATG-18-mediated cell non-autonomous signal depends on the release of neurotransmitters and neuropeptides

**Fig 6:**
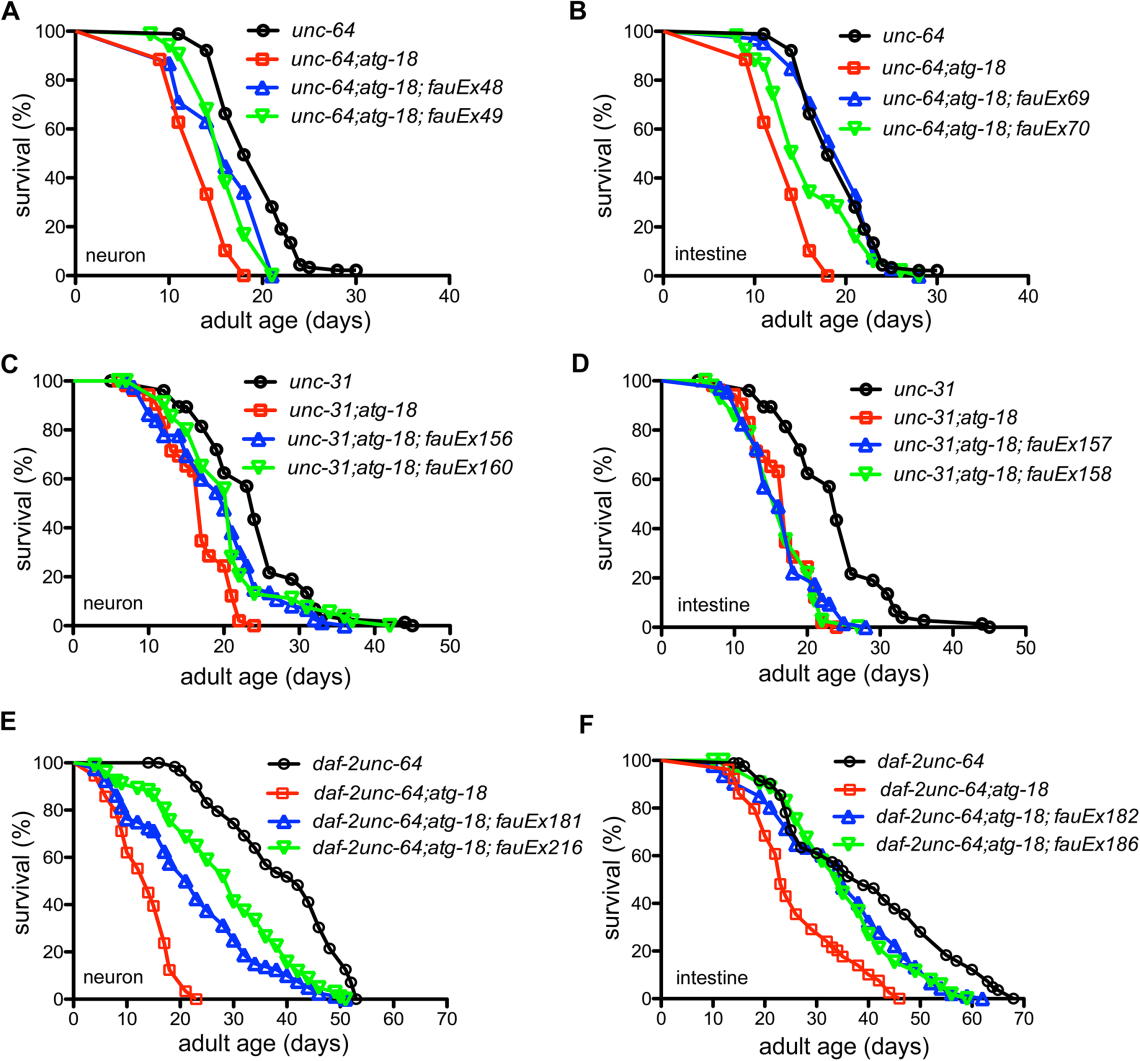
Neurotransmitters and neuropeptides are required for ATG-18-mediated cell non-autonomous signaling. Blockage of neurotransmitter release by the *unc-64(e246)* mutation suppresses the rescue effect of neuronal (A) and intestinal ATG-18 (B) on the lifespan of *atg-18(gk378)* mutants. Deficiency of neuropeptide secretion due to the *unc-31(e298)* mutation suppresses the rescue effect of neuronal (C) and intestinal ATG-18 (D) on the lifespan of *atg-18* mutants. *unc-64* mutations suppress the rescue effect of neuronal (E) and intestinal ATG-18 (F) on the lifespan of *daf-2;atg-18* mutants.

We investigated whether neurotransmitters and/or neuropeptides act downstream of ATG-18 to control *C*. *elegans* lifespan cell non-autonomously. Mutations in *unc-64*, the gene that encodes the worm ortholog of vertebrate syntaxin 1A, block the release of neurotransmitters [39]. We found the *atg-18* mutation decreases the lifespan of *unc-64(e246)* mutants (Fig 6, S10 Table). Expression of *atg-18* in neurons or intestinal cells only partially rescues the short lifespan of *unc-64;atg-18* (Fig 6A and 6B), which suggests neurotransmitters are required for neuronal and intestinal ATG-18 to maintain wildtype lifespan. To confirm the involvement of neurotransmitters in ATG-18-mediated neuronal signaling, we constructed *unc-13(e51);atg-18(gk378)* mutants. The *C*. *elegans* UNC-13 protein and its mammalian orthologs are important for normal neurotransmitter release. Mutations in *unc-13* block release of most neurotransmitters [40]. We found that the eggs of *unc-13;atg-18* mutants cannot hatch. Neuronal but not intestinal expression of *atg-18* can rescue this lethality, indicating neuronal ATG-18 influences neurotransmitter release.

We next examined the role of neuropeptides in autophagy-mediated cell non-autonomous signaling. UNC-31 is the *C*. *elegans* ortholog of human CADPS/CAPS (calcium-dependent activator protein). *unc-31* mutations block Ca^2+^-mediated release of dense core vesicles (DCVs) that contain neuropeptides [41, 42]. *unc-31(e298);atg-18(gk378)* mutants live shorter than *unc-31* mutants (P<0.0001, log-rank test) (Fig 6). Moreover, we found neuronal ATG-18 only partially rescues the short lifespan of *unc-31;atg-18* (P = 0.0004 and 0.0300) while intestinal ATG-18 has no effect (P = 0.6034 and 0.3698) (Fig 6C and 6D). As UNC-31 is exclusively expressed in neurons, intestinal ATG-18 should generate a signal to communicate with neurons that secrete neuropeptides to control lifespan.

We then examined the role of synaptic transmission in ATG-18-mediated cell non-autonomous signaling in *daf-2* mutants. We created *daf-2unc-64;atg-18* transgenic lines carrying either neuronal or intestinal expressed *atg-18*. Compared to the *daf-2unc-64* mutants, the *daf-2unc-64;atg-18* mutants live shorter (Fig 6). Both neuronal and intestinal expressed *atg-18* partially rescue the lifespan phenotype of *daf-2unc-64;atg-18* mutants (Fig 6E and 6F), indicating that UNC-64-mediated neurotransmitter release is required for ATG-18 to fully restore the lifespan of *daf-2unc-64;atg-18* mutants to the *daf-2unc-64* mutant level.

We next attempted to test the influence of UNC-31 on the rescue of *daf-2;atg-18* lifespan by neuronal or intestinal ATG-18. We found that *daf-2;unc-31;atg-18* mutant eggs cannot hatch and expression of *atg-18* in either neurons or intestinal cells cannot rescue this lethality. In the *daf-2unc-64;atg-18* mutant background, we also observed that the fertility is significantly reduced and only 18.99±2.98% (mean±s.e.m., n = 463) of eggs develop to adults. By contrast, 91.54±1.06% (mean±s.e.m., n = 1275) of *daf-2unc-64* eggs develop to adults in four days at 20°C. Interestingly, neuronal or intestinal expression of *atg-18* only partially rescues this developmental defect (for neuronal rescue, 38.36±3.99%, mean±s.e.m., n = 672; for intestinal rescue, 27.70±3.21%, mean±s.e.m., n = 240), which is consistent with the observation that UNC-64 is required for the rescue of *daf-2;atg-18* short lifespan by neuronal or intestinal ATG-18.

**Fig 7:**
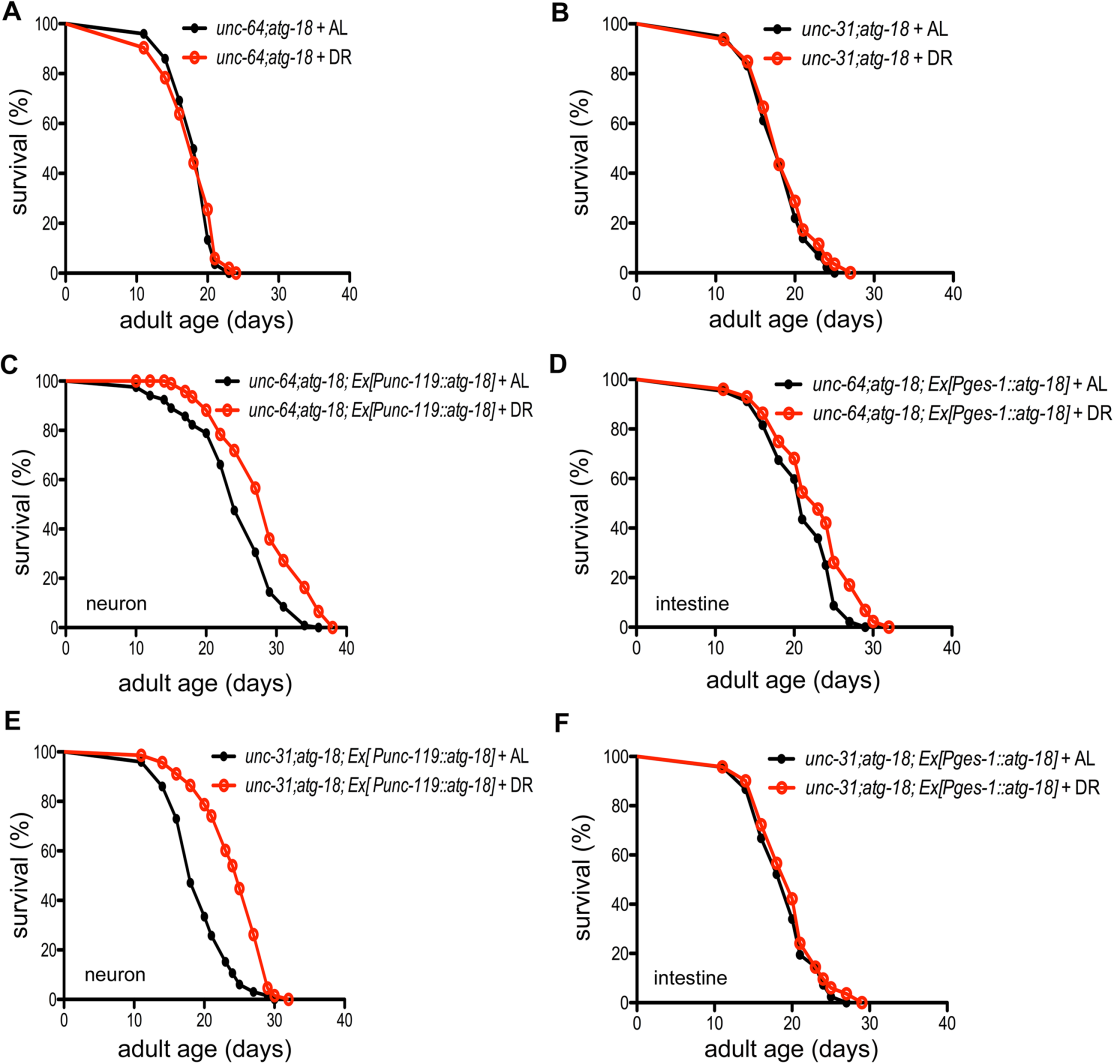
Neuropeptides are required for intestinal ATG-18-mediated cell non-autonomous signaling in DR response. (A, B) The *atg-18(gk378)* mutation suppresses the DR response in *unc-64* and *unc-31* mutants. (C, D) Expression of *atg-18* in neurons or intestinal cells confers response to DR in *unc-64* mutants. (E, F) The *unc-31(e298)* mutation does not block the rescue effect of neuronal ATG-18 on the DR response in *atg-18* mutants but fully suppresses the rescue effect of intestinal ATG-18.

Next, we tested if UNC-64 and UNC-31 are required for the cell non-autonomous regulation of *C*. *elegans* lifespan under sDR. We found that the lifespan of *unc-64* and *unc-31* mutants is increased after sDR treatment (S9 Fig, S11 Table) and *atg-18* mutations block this DR response (Fig 7A and 7B, S11 Table). Moreover, neuronal or intestinal expressed ATG-18 confers DR response in *unc-64;atg-18* mutants (Fig 7C and 7D), suggesting that neurotransmitter release might be dispensable for neuronal and intestinal ATG-18-mediated DR response. We also found that the *unc-31* mutation fails to block neuronal ATG-18-mediated DR response since the lifespan of *unc-31;atg-18* mutants carrying neuronal expressed ATG-18 is increased after DR treatment (Fig 7E). Interestingly, the *unc-31* mutation completely suppresses the intestinal ATG-18-mediated DR response (Fig 7F), which supports the cell non-autonomous function of intestinal ATG-18 in regulation of DR response.

Finally, to test whether ATG-18 influences the release of neurotransmitters we performed an aldicarb assay. Aldicarb inhibits acetylcholinesterase, preventing the breakdown of acetylcholine in the synaptic cleft, which causes paralysis in *C*. *elegans*. Gene mutations that block synaptic transmission render worms resistant to aldicarb-induced paralysis [43]. Similar to a previous report [39], while 100% of N2 worms become paralyzed after 3-hour treatment by 1mM aldicarb, none of *unc-64* mutants are paralyzed (S10 Fig). Interestingly *atg-18* mutants are as sensitive to aldicarb treatment as N2 worms (S10 Fig), indicating the *atg-18* mutation neither blocks acetylcholine release nor influences release of all neurotransmitters in general. This is consistent with the fact that sensory neurons, where ATG-18 acts, are generally not cholinergic [44]. Moreover, *unc-64;atg-18* mutants are resistant to aldicarb treatment indicating *unc-64* is downstream of ATG-18 in neurotransmitter signaling.

### Autophagy deficiency influences the expression levels of insulin-like peptide (ILP) genes

**Fig 8:**
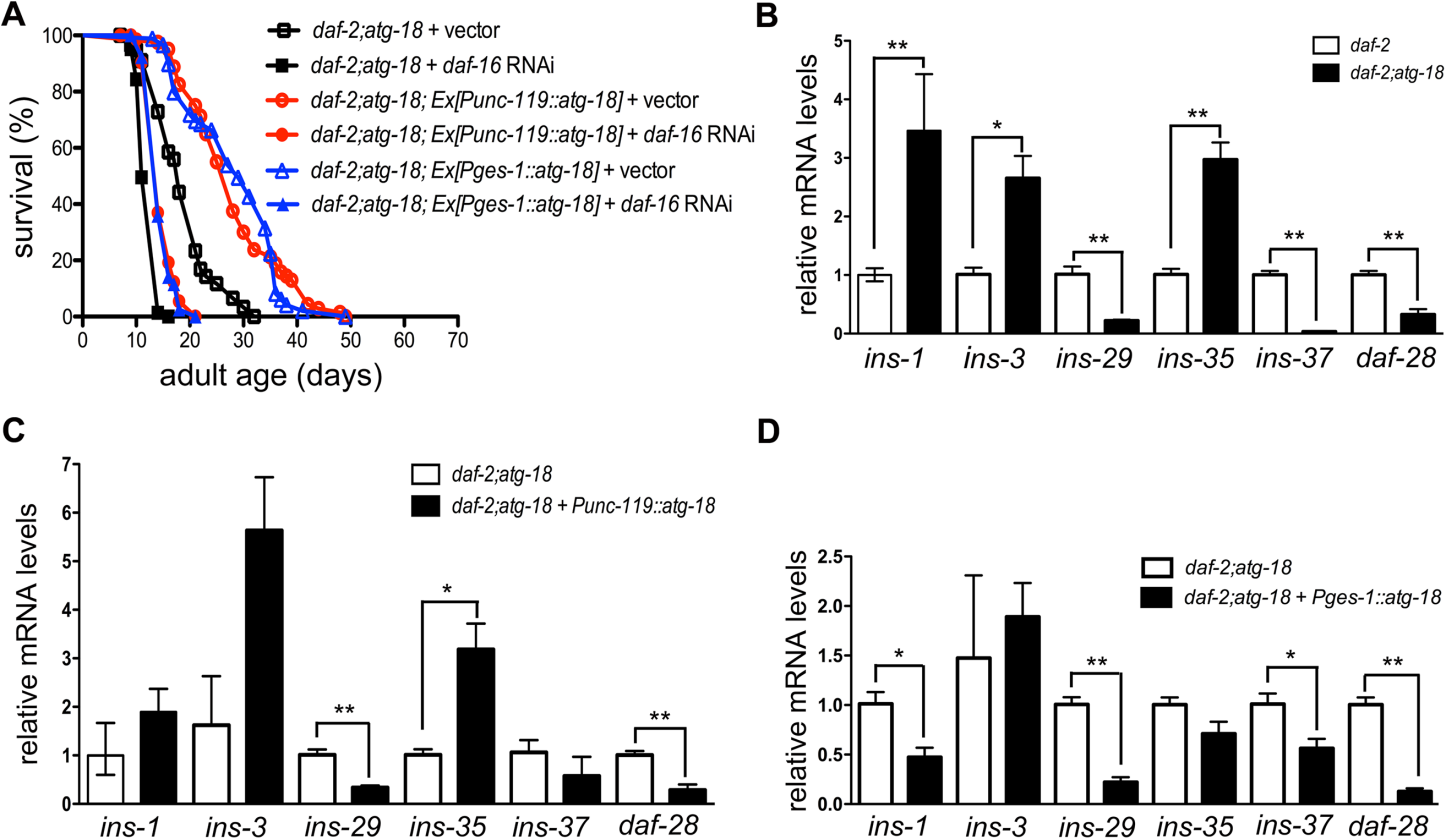
Autophagy deficiency influences the expression levels of insulin-like peptide genes. (A) Inhibition of DAF-16 by RNAi suppresses the rescue effect of neuronal and intestinal ATG-18 on the lifespan of *daf-2;atg-18* mutants. (B) The expression levels of *ins-1*, *ins-3* and *ins-35* are increased in *daf-2;atg-18* mutants, while the expression levels of *ins-29*, *ins-37* and *daf-28* are decreased. (C) Neuronal expression of *atg-18* fails to revert the change in expression of these six ILP genes in *daf-2;atg-18* mutants. (D) Intestinal ATG-18 reverts the expression of *ins-1* in *daf-2;atg-18* mutants but not *ins-29*, *ins-37* and *daf-28*. Asterisks indicate statistically significant differences relative to controls (* P < 0.05, ** P < 0.01, t-test). Details of the statistical analysis of the qPCR data is found in S13 Table.

DAF-16 is required for lifespan extension by inhibition of certain chemosensory neurons [13]. We found inhibition of DAF-16 blocks the rescue effect of either neuronal or intestinal ATG-18 on *daf-2;atg-18* lifespan (Fig 8A, S12 Table), which suggests both neuronal and intestinal ATG-18 act through DAF-16 to regulate *C*. *elegans* lifespan in response to reduced IGF signaling. Since DAF-16 is the major target for IGF signaling, we postulated that autophagy deficiency might influence the expression levels of ILP genes. We examined the expression levels of 40 ILP genes in *daf-2* and *daf-2;atg-18* mutants. We found that the expression levels of six ILP genes (*ins-1*, *ins-3*, *ins-29*, *ins-35*, *ins-37* and *daf-28*) are significantly changed in *daf-2;atg-18* mutants compared to those of *daf-2* mutants (Fig 8B, S13 Table). The expression levels of *ins-1*, *ins-3* and *ins-35* are increased in *daf-2;atg-18* mutants, while the expression levels of *ins-29*, *ins-37* and *daf-28* are decreased (Fig 8B, S13 Table). Then we tested if neuronal or intestinal expression of *atg-18* can revert the expression of these six ILPs genes in *daf-2;atg-18* mutants. We found that neuronal ATG-18 has no significant effect on the expression levels of *ins-1* (p = 0.1038 and 0.2315 for two trials), *ins-3* (p = 0.0548 and 0.74513 for two trials) and *ins-37* (p = 0.3611 and 0.6729 for two trials). Moreover, contradictory to our prediction, neuronal ATG-18 further increases the expression levels of *ins-35* and further decreases the expression levels of *ins-29*, and *daf-28* in *daf-2;atg-18* mutants (Fig 8C). These data indicate that neuronal expression of *atg-18* fails to revert the change in expression of six ILP genes in *daf-2;atg-18* mutants.

We also found that intestinal ATG-18 does not influence the expression of *ins-3* and *ins-35* (Fig 8D, S13 Table). However, it significantly decreases the expression of *ins-1*, *ins-29*, *ins-37* and *daf-28* (Fig 8D, S13 Table). Since the expression level of *ins-1* is increased in *daf-2;atg-18* mutants compared to that of *daf-2* mutants, this data indicate that intestinal ATG-18 reverts the expression of *ins-1* in *daf-2;atg-18* mutants but not *ins-29*, *ins-37* and *daf-28*.

## Discussion

**Fig 9:**
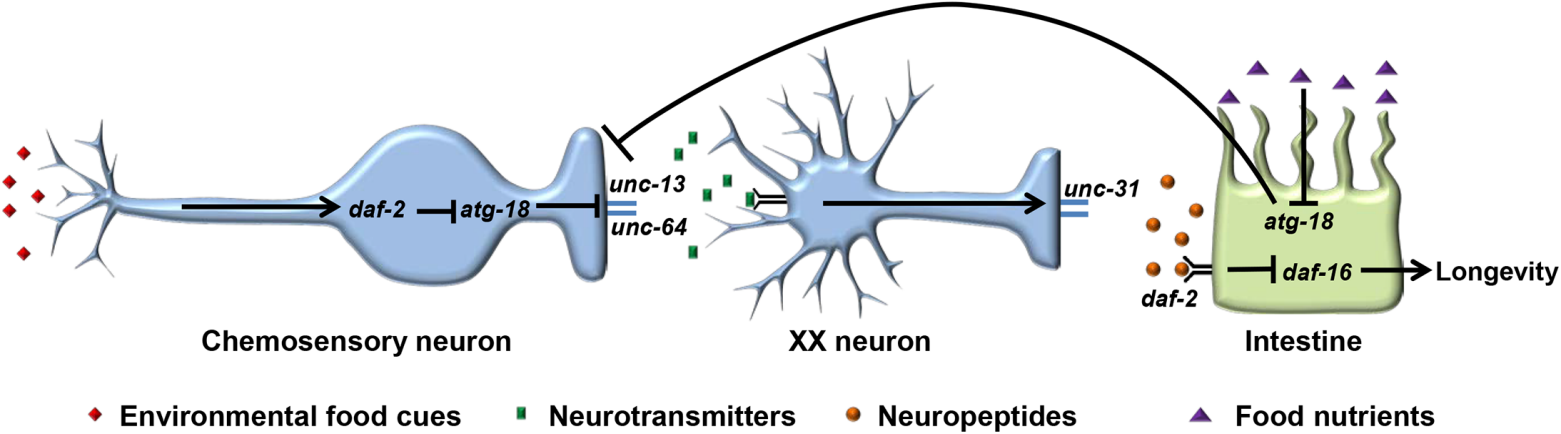
A model depicting the role of ATG-18 in neuroendocrine regulation of *C*. *elegans* lifespan. Neuronal and intestinal ATG-18 acts in parallel to respond to environmental food cues and assimilated food nutrients, respectively. This food response inhibits the function of unidentified XX neurons that secrete neuropeptides which negatively regulate *C*. *elegans* lifespan. See text for details. Proposed wildtype functions are shown, with arrows indicating stimulation of activities and T-bars indicating inhibition.

Autophagy is required for IGF signaling and DR to extend lifespan in *C*. *elegans* [30, 31, 33]. However, the mechanisms are not well understood. Here we show that autophagy is an essential component in the neuroendocrine regulation of *C*. *elegans* lifespan. The DAF-2 insulin-like receptor is highly expressed in neurons, as detected by an anti-DAF-2 antibody, and the *daf-2* promoter is active in both neurons and intestinal cells [45, 46]. Apfeld and Kenyon found that neuronal DAF-2 acts cell non-autonomously to regulate longevity [47], while Wolkow et al. reported that DAF-2 expressed in either neurons or intestinal cells prevents the long lifespan of *daf-2* mutants [48]. Interestingly, DAF-16, the major target of DAF-2, primarily functions in the intestine to mediate DAF-2 regulated longevity while neuronal DAF-16 is dispensable [49]. This suggests DAF-2 can work in both neurons and intestinal cells while DAF-16 acts mainly in the intestine to control lifespan. Autophagy is induced in *daf-2* mutants and acts downstream of DAF-2 in a DAF-16 independent manner [31, 33]. Therefore, it is reasonable to speculate that DAF-2 regulates autophagy in neurons. Our data indicate ATG-18 in chemosensory neurons and intestine acts in parallel to mediate the influence of DAF-2 on lifespan through DAF-16. Since autophagy is highly sensitive to nutrients, we propose neuronal and intestinal autophagy responds to environmental food cues and assimilated food nutrients respectively to regulate *C*. *elegans* lifespan in response to reduced IGF signaling (Fig 9). In this model, environmental food cues inhibit autophagy though DAF-2 in chemosensory neurons. Autophagy negatively influences the availability of neurotransmitters that require UNC-64 for release. These neurotransmitters inhibit the function of unidentified (herein XX) neurons which secrete neuropeptides that negatively influence *C*. *elegans* lifespan by inhibition of DAF-16. Indeed, DAF-16 is required for the lifespan extension resulting from mutations in odor receptors or ablation of food sensory neurons [12, 13]. In parallel to the neuronal regulation of autophagy by environmental food cues, food nutrients absorbed by the intestine also inhibit autophagy. In the absence of this inhibition, autophagy promotes longevity by modulating the function of chemosensory neurons. According to this model, the change of physiological levels of insulin peptides due to autophagy deficiency enhances DAF-2 activity and suppresses the *daf-2* longevity phenotype. This model is supported by the following experimental data in the present study. Firstly, both neuronal and intestinal expressed *atg-18* only partially rescue the short lifespan of *daf-2unc-64;atg-18* mutants, which indicates that UNC-64-mediated neurotransmitter release is required for ATG-18 to fully restore the lifespan of *daf-2unc-64;atg-18* mutant to the *daf-2unc-64* mutant level. Secondly, the fertility of *daf-2unc-64;atg-18* mutants is significantly reduced and neuronal or intestinal expression of *atg-18* only partially rescues this developmental defect. This is consistent with the observation that UNC-64 is required for the rescue of *daf-2;atg-18* short lifespan by neuronal or intestinal ATG-18. Thirdly, although *unc-13(e51)* is viable, the *unc-13(e51);atg-18(gk378)* double mutant is lethal. Additionally, it has been reported that severe *unc-13* mutants are lethal [50]. If *unc-13* and *atg-18* are in a parallel pathway, we expect that either neuronal or intestinal ATG-18 should rescue the lethality as expression of *atg-18* in these two tissues fully rescues the short lifespan of *atg-18* mutants. However, we found that only neuronal ATG-18 can rescue the lethality, which indicates that UNC-13 and ATG-18 act in a linear pathway in neurons and UNC-13 is downstream of ATG-18. Fourthly, because the *daf-2;unc-31;atg-18* mutant is lethal we could not test the requirement of UNC-31 for neuronal or intestinal ATG-18 to rescue the short lifespan of *daf-2;atg-18*. However, UNC-31 is necessary for neuronal or intestinal ATG-18 to rescue the short lifespan of *atg-18*. Moreover, neuronal or intestinal ATG-18 fails to rescue the lethality of *daf-2;unc-31;atg-18* mutants, indicating that secreted neuropeptides are the final target of neuronal and intestinal ATG-18. Finally, inhibition of *daf-16* by RNAi completely blocks the ability of neuronal or intestinal ATG-18 to rescue the lifespan phenotype of *daf-2;atg-18* mutants, indicating DAF-16 is the target regulated by the secreted neuropeptides.

Our model is supported by a recent study showing that ILPs secreted by chemosensory neurons facilitated by UNC-31 enable L1 *C*. *elegans* larva to survive from starvation via DAF-16 [51]. Indeed, autophagy is essential for survival of animals under starvation conditions [18]. Therefore, it is reasonable to speculate that neuronal insulin-like peptides act downstream of neuronal and intestinal ATG-18 to regulate *C*. *elegans* lifespan. *C*. *elegans* encodes 40 ILPs most of which are not characterized [52]. Some of ILPs are agonists for DAF-2 while others are antagonists [52]. A recent study indicates that the nervous system secretes a specific group of ILPs and that the agonistic-antagonistic ILP balance determines the activity of intestinal DAF-2 during the larval development in response to environmental food availability [53]. Thus, autophagy may influence the physiological levels of both agonist and antagonist ILPs from XX neurons instead of only agonist ILPs as shown in the model. Interestingly, although most of the ILPs are expressed in neurons, some are expressed in the hypodermis [54], which may explain our data showing that expression of ATG-18 in the hypodermis fully rescues *daf-2;atg-18* lifespan. However hypodermal ATG-18 has no influence on wildtype lifespan.

We performed qPCR to measure the expression levels of 40 ILPs and found that the expression levels of *ins-1*, *ins-3* and *ins-35* are increased in *daf-2;atg-18* mutants, while the expression levels of *ins-29*, *ins-37* and *daf-28* are decreased compared to those of *daf-2* mutants. DAF-28, INS-3 and INS-35 are classified as agonists for DAF-2 and INS-1 is an antagonist [55, 56]. The interaction of INS-29 and INS-35 with DAF-2 has not been reported. If neuronal ATG-18 acts through these ILPs, we expect that expression of neuronal ATG-18 in *daf-2;atg-18* mutants will revert the expression of these genes to the *daf-2* levels. However, this is not our observation. There are two possible interpretations for this result. It is possible that insulin peptides are not involved in ATG-18 mediated cell non-autonomous signaling. Alternatively, *atg-18* mutations influence the physiological levels of certain insulin peptides that regulate intestinal DAF-16 activity. However, this influence fails to revert the transcription levels of the six aforementioned ILPs genes to *daf-2* levels. We favor this possibility because both neuronal and intestinal autophagy act though DAF-16 and intestinal DAF-16 acts downstream of DAF-2 signaling to regulate *C*. *elegans* lifespan [49]. A future study can be performed to systematically examine the production and secretion of all 40 insulin peptides in *daf-2;atg-18* mutants, which might help to determine whether insulin peptides are involved in neuronal ATG-18 mediated cell non-autonomous signaling.

Interestingly, we found that intestinal ATG-18 reverts the expression of *ins-1* in *daf-2;atg-18* mutants to the *daf-2* level, suggesting that INS-1 is a candidate insulin peptide that may be involved in cell non-autonomous regulation of lifespan by intestinal ATG-18. INS-1 is most related to mammalian insulin [55]. It functions redundantly with other insulin-like peptides to regulate reproductive growth and lifespan [55]. GFP expression under the control of the *ins-1* promoter is detected in some amphid sensory neurons including ASI and ASJ which regulate dauer formation [55]. Dauer larva is a dispersal stage of *C*. *elegans* that forms under starvation conditions and INS-1 promotes dauer entry [55, 57]. A functional INS-1::GFP reporter gene is strongly expressed in sensory neurons but not in intestinal cells [53]. Interestingly INS-1 has been reported to act on intestinal DAF-2 to promote dauer formation through DAF-16 [53]. Our data show that intestinal ATG-18 generates cell non-autonomous signal molecules that communicate with neurons to regulate lifespan. Thus, INS-1 could be a candidate neuropeptide secreted by neurons in response to this communication. In support of our data, a crosstalk between neuronal and intestinal autophagy has been reported to influence *Drosophila* lifespan, which is linked to altered ILP levels [58].

Previous studies indicate that DR can act in parallel to IGF signaling to increase *C*. *elegans* lifespan and that DAF-16 is not required for lifespan extension under certain DR protocols [5]. We found that ATG-18 in chemosensory neurons and intestinal cells enables *atg-18* mutants to respond to DR. Moreover, we found that neurotransmitters may not be required for neuronal ATG-18 to mediate the DR response as the *unc-64(e246)* mutation does not block the lifespan extension of *atg-18* mutants conferred by neuronal expression of ATG-18 under DR treatment. However, since the *unc-31(e298)* mutation completely block the DR response of *atg-18* mutants carrying intestinal ATG-18, neuropeptides act downstream of intestinal ATG-18 to mediate the DR response. In addition, we found that ATG-18 in muscles plays a minor role in maintaining *C*. *elegans* wildtype lifespan and response to reduced IGF signaling but ATG-18 in the muscles or hypodermis has no contribution to the DR response. These results indicate that ATG-18 in individual tissues has different roles in regulating *C*. *elegans* lifespan under specific conditions.

Recently, Gelino et al. reported that intestinal knockdown of *atg-18* shortens the lifespan of the DR mutant *eat-2*, demonstrating that under this DR condition neuronal *atg-18* cannot sufficiently mediate the DR response [59]. By contrast, our data show that neuronal ATG-18 confers the DR response in *atg-18* mutants. This discrepancy might be caused by the difference of DR protocols used in these two studies. Indeed, while DAF-16 is required for the sDR response [32], it is dispensable for the longevity phenotype of *eat-2* mutants [60]. Nevertheless, intestinal ATG-18 clearly is essential for lifespan extension in response to DR under these two different DR protocols.

ATG-18 and its mammalian homolog, WIPI1 and WIPI2, are essential for the autophagy process [20–22]. Our data show that *atg-18* mutations suppress autophagy induction in *daf-2* mutants and wildtype animals treated by DR, indicating that the autophagy function of ATG-18 controls *C*. *elegans* lifespan. Since autophagy is evolutionarily conserved from *C*. *elegans* to mammals, the cell non-autonomous function of autophagy in the chemosensory neurons and intestine to control aging via a neuroendocrine mechanism may be conserved.

## Materials and methods

### Strains

To obtain the *daf-2(1370);atg-18(gk378)* double mutant, *daf-2(e1370);dpy-11(e224)* was constructed. N2 males were crossed to the *daf-2;dpy-11* hermaphrodites to obtain *daf-2/+; dpy-11/+* males that were crossed to *atg-18* hermaphrodites to obtain worms with genotype *daf-2/+; dpy-11/atg-18*. The strain *daf-2;dpy-11/atg-18* was isolated based the Daf-c phenotype of *daf-2* mutants at 25°C. *daf-2;atg-18* mutant strain was isolated from progeny of *daf-2;dpy-11/atg-18*. To construct *unc-64(e246);atg-18(gk378)*, *unc-64/+* males were crossed to *atg-18* hermaphrodites. The *unc-64;atg-18* mutants were isolated from progeny of *unc-64/+;atg-18/+*. The same strategy was used to construct *unc-31(e298);atg-18(gk378)*. The *unc-13(e51);atg-18(gk378)* mutant was constructed by crossing N2 males with *unc-13;dpy-11* hermaphrodites. The *unc-13/+;dpy-11/+* males were crossed to *atg-18* hermaphrodites to obtain *unc-13/+; dpy-11/atg-18* worms. The strain *unc-13;dpy-11/atg-18* was isolated. To construct *daf-2(e1370)unc-64(e246);atg-18(gk378)*, N2 males were crossed to the *daf-2unc-64* hermaphrodites to obtain *daf-2/+unc-64/+* males that were crossed to *atg-18* hermaphrodites to obtain worms with genotype *daf-2/+unc-64/+; dpy-11/atg-18*. The strain *daf-2unc-64;atg-18* was isolated. A similar strategy was used to construct *daf-2(e1370);unc-31(e298); dpy-11(e224)/atg-18(gk378)*. The homozygous deletion mutation of *atg-18* in all strains was confirmed by a standard single-worm PCR.

Microinjection was used to generate the extrachromosomal (*Ex*) array transgenic lines by injecting the corresponding plasmid (50 ng/μl) with either the pRF4 *rol-6(su1006)* marker plasmid (100 ng/μl) or the *Pmyo-2*::*gfp* marker (50 ng/μl): N2; Ex[*Patg-18*::*atg-18 + rol-6(su1006)]*, N2; Ex[*rol-6(su1006)]*, *atg-18; Ex[Patg-18*::*atg-18 + rol-6(su1006)]*, *atg-18; Ex[Punc-119*::*atg-18 + rol-6(su1006)]*, *atg-18; Ex[Pges-1*::*atg-18 + rol-6(su1006)]*, *atg-18; Ex[Pmyo-3*::*atg-18 + rol-6(su1006)]*, *atg-18; Ex[Pdpy-7*::*atg-18 + rol-6(su1006)]*, *atg-18; Ex[Pgpa-3*::*atg-18 + rol-6(su1006)]*, *daf-2;atg-18; Ex[Patg-18*::*atg-18 + rol-6(su1006)]*, *daf-2;atg-18; Ex[Punc-119*::*atg-18 + rol-6(su1006)]*, *daf-2;atg-18; Ex[Pges-1*::*atg-18 + rol-6(su1006)]*, *daf-2;atg-18; Ex[Pmyo-3*::*atg-18 + rol-6(su1006)]*, *daf-2;atg-18; Ex[Pdpy-7*::*atg-18 + rol-6(su1006)]*, *daf-2;atg-18; Ex[Prgef-1*::*atg-18 + rol-6(su1006)]*, *daf-2;atg-18; Ex[Pgpa-3*::*atg-18 + rol-6(su1006)]* (*fauEx88*, *fauEx89*, *fauEx90* and *fauEx91*), *daf-2;atg-18; Ex[Pdaf-11*::*atg-18 + rol-6(su1006)]* (*fauEx119* and *fauEx122*), *daf-2;atg-18; Ex[Punc-42*::*atg-18 + rol-6(su1006)]* (*fauEx115* and *fauEx116*), *daf-2;atg-18; Ex[Podr-2*::*atg-18 + rol-6(su1006)]* (*fauEx101* and *fauEx102*), *daf-2;atg-18; Ex[Podr-10*::*atg-18 + rol-6(su1006)]* (*fauEx82* and *fauEx83*), *daf-2;atg-18; Ex[Ptph-1*::*atg-18 + rol-6(su1006)]* (*fauEx86* and *fauEx87*), *daf-2;atg-18; Ex[Psrh-220*::*atg-18 + rol-6(su1006)]* (*fauEx85* and *fauEx124*), *daf-2;atg-18; Ex[Podr-1*::*gfp*::*atg-18 + rol-6(su1006)]*, *daf-2;atg-18; Ex[Podr-10*::*gfp*::*atg-18 + rol-6(su1006)]*, *daf-2;atg-18; Ex[Ptph-1*::*gfp*::*atg-18 + rol-6(su1006)]*, *daf-2;atg-18; Ex[Psrh-220*::*gfp*::*atg-18 + rol-6(su1006)]*, *unc-64;atg-18; Ex[Punc-119*::*atg-18 + Pmyo-2*::*gfp]* (*fauEx48* and *fauEx49*), *unc-64;atg-18; Ex[Pges-1*::*atg-18 + Pmyo-2*::*gfp]* (*fauEx69* and *fauEx70*), *unc-31;atg-18; Ex[Punc-119*::*atg-18 + Pmyo-2*::*gfp]* (*fauEx56* and *fauEx60*), *unc-31;atg-18; Ex[Pges-1*::*atg-18 + Pmyo-2*::*gfp]* (*fauEx157* and *fauEx158*), *unc-13;dpy-11/atg-18; Ex[Punc-119*::*atg-18 + Pmyo-2*::*gfp]*, *unc-13;dpy-11/atg-18; Ex[Pges-1*::*atg-18 + Pmyo-2*::*gfp]*, *daf-2unc-64;atg-18; Ex[Punc-119*::*atg-18 + Pmyo-2*::*gfp]*, *daf-2unc-64;atg-18; Ex[Pges-1*::*atg-18 + Pmyo-2*::*gfp]*, *daf-2;unc-31; dpy-11/atg-18; Ex[Punc-119*::*atg-18 + Pmyo-2*::*gfp]*, *daf-2;unc-31; dpy-11/atg-18; Ex[Pges-1*::*atg-18 + Pmyo-2*::*gfp]*, *daf-2unc-64;atg-18; Ex[Punc-119*::*atg-18 + Pmyo-2*::*gfp]*, *daf-2unc-64;atg-18; Ex[Pges-1*::*atg-18 + Pmyo-2*::*gfp]*.

To examine autophagy activity in hypodermal seam cells, the following transgenic lines were created by injecting the corresponding plasmid (50 ng/μl) with both *Plgg-1*::*gfp*::*lgg-1* (50 ng/μl) and the pRF4 *rol-6(su1006)* marker plasmid (100 ng/μl): *atg-18; Ex[Plgg-1*::*gfp*::*lgg-1+ rol-6(su1006)]*, *atg-18; Ex[Punc-119*::*atg-18* + *Plgg-1*::*gfp*::*lgg-1+ rol-6(su1006)]*, *atg-18; Ex[Pges-1*::*atg-18* + *Plgg-1*::*gfp*::*lgg-1+ rol-6(su1006)]*, *atg-18; Ex[Pdpy-7*::*atg-18* + *Plgg-1*::*gfp*::*lgg-1+ rol-6(su1006)]*, *atg-18; Ex[Pmyo-3*::*atg-18* + *Plgg-1*::*gfp*::*lgg-1+ rol-6(su1006)]*, *daf-2;atg-18; Ex[Plgg-1*::*gfp*::*lgg-1+ rol-6(su1006)]*, *daf-2;atg-18; Ex[Punc-119*::*atg-18* + *Plgg-1*::*gfp*::*lgg-1+ rol-6(su1006)]*, *daf-2;atg-18; Ex[Pges-1*::*atg-18* + *Plgg-1*::*gfp*::*lgg-1+ rol-6(su1006)]*, *daf-2;atg-18; Ex[Pdpy-7*::*atg-18* + *Plgg-1*::*gfp*::*lgg-1+ rol-6(su1006)]*, *daf-2;atg-18; Ex[Pmyo-2*::*atg-18* + *Plgg-1*::*gfp*::*lgg-1+ rol-6(su1006)]*. The *Plgg-1*::*gfp*::*lgg-1* plasmid, N2 and *daf-2(e1370)* transgenic animals carrying GFP::LGG-1 marker were gifted by Dr. Beth Levine at UT Southwestern Medical Center.

### Molecular cloning

Genomic DNA was extracted from N2 animals and used as a template to amplify the following DNA fragments: 3kb *atg-18* promoter, 1.6kb full-length *atg-18* genomic DNA, 2.2kb *unc-119* promoter, 3.3kb *ges-1* promoter, 2.5kb *myo-3* promoter, 344bp *dpy-7* promoter, 4.2kb *daf-11* promoter, 2.6kb *unc-42* promoter, 5.9kb *gpa-3* promoter, 2.6kb *odr-2* promoter, 1.2kb *odr-10* promoter, 3.1kb *tph-1* promoter and 2.1kb *srh-220* promoter. The following DNA fragments were amplified with Advantage 2 Polymerase (Clontech, *Mountain View*, *CA*), cloned into pGEMT vector (Promega, *Madison*, *WI*) and sequenced: *atg-18* genomic DNA, *Punc-119*, *Pdpy-7*, *Pdaf-11*, *Pgpa-3*, *Podr-10* and *Psrh-220*. For the *atg-18* genomic DNA, the whole gene was sequenced to confirm there were no PCR errors introduced. The rest of the promoter DNA fragments were amplified with Vent DNA polymerase (New England BioLabs, *Ipswich*, *MA*), cloned into the pSCB vector using the StrataClone PCR Cloning Kit (Agilent Technologies, Inc., *Santa Clara*, *CA*) and sequenced.

To construct the plasmid *Patg-18*::*atg-18*, the *atg-18* genomic DNA was excised with XmaI and KpnI, and cloned into the *C*. *elegans* expression vector L754. The 3kb *atg-18* promoter was cloned into SphI and XmaI sites right upstream of *atg-18* gene. To construct plasmids with tissue-specific *atg-18* expression, the *atg-18* promoter was excised with SphI and XmaI restriction enzymes, and replaced with *Punc-119*, *Pges-1*, *Pdpy-7*, *Pdaf-11* and *Punc-42*, respectively. To construct the *Pmyo-3*::*atg-18* plasmid, the *myo-3* promoter was excised from pSCB vector with NheI and KpnI and cloned into L754. The full-length *atg-18* genomic DNA was amplified with primers bearing restriction enzyme sites KpnI and SacI, respectively, cloned into pGEMT and sequenced. The *atg-18* genomic DNA was excised with KpnI and SacI, and inserted downstream of the *myo-3* promoter in L754 vector. The following plasmids with tissue-specific *atg-18* expression were constructed by replacing the *myo-3* promoter with corresponding promoter fragments: *Pgpa-3*::*atg-18*, *Podr-2*::*atg-18*, *Podr-10*:*atg-18*, *Ptph-1*::*atg-18* and *Psrh-220*::*atg-18*. All plasmids were sequenced to confirm the junction between the promoter and the beginning of the *atg-18* gene. Plasmid DNA was prepared for microinjection by using Zymo Plasmid Miniprep Kit (Zymo Research, *Irvine*, *CA*). Promoters active in different chemosensory neurons are: *Pdaf-11* in ASE, ASI, ASJ, ASK, AWB, AWC [61]; *Punc-42* in ASH [62]; *Pgpa-3* in ADF, ADL, ASE, ASG, ASH, ASI, ASJ, ASK, AWA, AWC [63, 64]; *Podr-10* in AWA [36], *Podr-2* in ASG [38]; *Ptph-1* in ADF [37] and *Psrh-220* in ADL [36]. Some promoters are also active in other non-amphid neurons that are not listed here.

To construct the *Podr-2*::*gfp*::*atg-18* reporter gene, the *gfp* coding sequence was amplified from the pPD95.75 plasmid. The *gfp* fragment flanked by the KpnI site at both ends was cloned into pGEMT and sequenced to confirm that there are no mutations in the *gfp* sequence. The *gfp* fragment was then inserted downstream of the *odr-2* promoter and upstream of the *atg-18* genomic DNA. The orientation of the *gfp* sequence was confirmed by sequencing and the *gfp* sequence was fused in frame with *atg-18* gene. A similar strategy was used to construct the following three plasmids: *Podr-10*::*gfp*::*atg-18*, *Ptph-1*::*gfp*::*atg-18*, *Psrh-220*::*gfp*::*atg-18*.

### Lifespan experiment

Animals were grown on NG agar plates seeded with *E*. *coli* strain OP50. Well-fed L4 hermaphrodites were picked up for lifespan experiments on day zero. OP50 bacteria were grown overnight at 37°C with shaking. For the lifespan experiments using solid dietary restriction (sDR) assay [32], the overnight culture was washed and re-suspended in S Basal media without cholesterol. Serial dilutions were performed to achieve bacterial concentrations of 5.0 X 10^11^ cells/ml (AL: *ad libitum*) and 5.0 X 10^9^ cells/ml (DR: dietary restriction). 250 μl of these diluted bacterial cultures was spotted on each 60 mm sDR agar plate on the day of transfer. sDR plates were made by modifying the standard NGM plate formula to exclude peptone, increase agar concentration to 2.0% and include ampicillin (50μg/mL). L4 larval stage worms were picked up from animals grown on regular OP50 food plates. On the next day, designated day 1, adults were placed on fresh sDR plates with 30 worms on each plate. Three plates were prepared and there were 90 worms total for each group at the beginning of experiments.

For the *daf-16* RNAi lifespan experiment, RNAi feeding was performed as described by Kamath et al [65]. Briefly, L4 hermaphrodites were put on RNAi or control plates for thirty-six hours. The worms were then transferred to fresh RNAi or control plates and allowed to lay eggs. The L4 hermaphrodites of the RNAi-treated progeny were collected for experiments.

All lifespan experiments were performed at 20°C; animals were transferred to fresh plates every day during the reproductive period, and every other day thereafter. Animals were scored as dead when they failed to respond to touch. Animals that died from internal hatching or vulval rupture were censored from statistical analysis. GraphPad Prism 5 (GraphPad Software, *La Jolla*, *CA*) was used to generate survival curves, to obtain the median survival times in each group and to perform log-rank survival analyses (Kaplan-Meier method).

### qRT-PCR

Worms were allowed to grow on OP50 food for 3 days. These animals were harvested from the plate and washed with M9 buffer to remove all bacteria on the third day. RNA extraction was then done using the Trizol kit (Zymo). First strand cDNA was synthesized from the RNA samples by using the SuperScript First-Strand Synthesis System (Invitrogen). The expression levels of the ILPs were measured in triplicate using the SYBR green dye (Quanta) on an ABI Prism 7000 real-time PCR machine (Applied Biosystems). The relative-fold changes were calculated using the 2^−ΔΔCT^ method. The entire experiment was repeated at least once with RNA extracted from new samples to confirm the original results. The primer sequence used for qRT-PCR was derived from a previous publication [66].

### Autophagy activity analysis

Autophagy activity was analyzed by counting autophagosomes that appear as fluorescence puncta in hypodermal seam cells [33, 35]. Animals were grown at 20°C on OP50 or sDR food plates (preparation of sDR and control food plates were described in the above Lifespan Experiment section). L3-L4 larva were collected, mounted on agar pad slides and observed under a Zeiss upright fluorescence microscope (Axio Imager A2). Pictures were captured using the Zeiss AxioCam digital camera at 1,000X magnification. Approximately 20 animals were examined for each strain in each experiment.

### Aldicarb assay

The experiment was performed as described previously [43]. In brief, L4 worms were picked and allowed to develop to adults on OP50 food plates. On the following day, day 1, adults were transferred to freshly prepared aldicarb plates to begin the assay. The aldicarb plates were prepared on the day of the experiment by spreading aldicarb (Sigma) on NGM plates to achieve a concentration of 1mM. A spot of OP50 was added to the center of the plates to keep the worms concentrated. Each strain was tested in triplicate with 20 worms per plate. Paralysis of animals was scored thirty minutes after the worms were transferred to the aldicarb plates. Worms were considered paralyzed once they showed no movement in response to being prodded three times by a platinum wire. GraphPad Prism 5 was used to generate the graph and to perform statistical analysis.

## Supporting information

S1 Fig Overexpression of *atg-18* under the control of its native promoter decreases the lifespan of wildtype N2 animals. (TIF)

S2 Fig The *atg-18(gk378)* mutation significantly decreases the lifespan of *daf-2(e1370)* mutants (A) and muscle ATG-18 modestly increases the lifespan of *daf-2(e1370)*;*atg-18(gk378)* mutants (B).
(TIF)

S3 Fig Expression of *atg-18* driven by the pan-neuronal promoter *rgef-1* fully restores the lifespan of *daf-2;atg-18* mutants to the *daf-2(e1370)* level. (TIF)

S4 Fig Representative pictures of autophagosomes in seam cells. (A) N2 (B) *daf-2(e1370)* (C) *atg-18(gk378)* (D) *daf-2;atg-18* (E) *daf-2;atg-18; Ex[Punc-119*::*atg-18]* (F) *daf-2;atg-18; Ex[Pges-1*::*atg-18]* (G) *daf-2;atg-18; Ex[Pdpy-7*::*atg-18]* (H) *daf-2;atg-18; Ex[Pmyo-3*::*atg-18]* (I) N2 +AL (J) N2 + DR (K) *atg-18* + AL (L) *atg-18* + DR (M) *atg-18;Ex[Punc-119*::*atg-18]* + AL (N) *atg-18;Ex[Punc-119*::*atg-18]* + DR (O) *atg-18;Ex[Pges-1*::*atg-18]* + AL (P) *atg-18;Ex[Pges-1*::*atg-18]* + DR (Q) *atg-18;Ex[Pdpy-7*::*atg-18]* + AL (R) *atg-18;Ex[Pdpy-7*::*atg-18]* + DR (S) *atg-18;Ex[Pmyo-3*::*atg-18]* + AL (T) *atg-18;Ex[Pmyo-3*::*atg-18]* + DR. Arrows denote representative autophagosomes. Scale bars: 10μM.
(TIF)

S5 Fig Expression of *atg-18* in ASH neurons (*Punc-42*::*atg-18*) (A) and in ASE, ASI, ASJ, ASK, AWB and AWC chemosensory neurons (*Pdaf-11*::*atg-18*) (B) has no obvious effect on the lifespan *daf-2(e1370)*;*atg-18(gk378)* mutants.
(TIF)

S6 Fig Expression of the *gfp*::*atg-18* reporter gene under the control of neuron-specific promoters. (A) The *odr-2* promoter drives expression of the *gfp*::*atg-18* reporter gene in ASG chemosensory neurons, the nerve ring and other neurons (see text for details). (B, C) The *odr-10* and *srh-220* promoters drive the expression of *gfp*::*atg-18* in AWA and ADL chemosensory neurons, respectively. (D, E) Expression of *gfp*::*atg-18* in ADF, NSM and HSN serotonergic neurons under the control of the *tph-1* promoter. The anterior of the worm body in the image points to the left. Scale bars: 10μM.
(TIF)

S7 Fig Expression of *atg-18* in chemosensory neurons increases the lifespan of *atg-18* mutants. (TIF)

S8 Fig Expression of *atg-18* in chemosensory neurons (*Pgpa-3*::*atg-18*) including food sensory neurons restores the DR response in *atg-18(gk378)* mutants. (TIF)

S9 Fig Dietary restriction extends the lifespan of *unc-64* (A) and *unc-31* (B) mutants.
(TIF)

S10 Fig The *atg-18(gk378)* mutation has no influence on release of the neurotransmitter acetylcholine measured by resistance to aldicarb-induced paralysis. The paralysis was examined in triplicates for each sample at the following time points after drug treatment: 0 min, 100 min, 140 min, 160 min and 180 min. t-test was performed for statistical analysis at each time point. The p value at 100-minute time point is: 0.3210 for N2 v.s. *atg-18*, 0.5614 for *unc-64* v.s. *unc-64;atg-18* and <0.0001 for *atg-18* v.s. *unc-64;atg-18*. Similar P values are obtained at each time point.
(TIF)

S1 Table Statistical analysis of lifespan data for Fig 1. (DOCX)

S2 Table Statistical analysis of lifespan data for S1 Fig. (DOCX)

S3 Table Statistical analysis of lifespan data for Fig 2. (DOCX)

S4 Table Statistical analysis of lifespan data for Fig 3 and S2 Fig. (DOCX)

S5 Table Statistical analysis of lifespan data for S3 Fig (DOCX)

S6 Table Statistical analysis of autophagy induction. (DOCX)

S7 Table Statistical analysis of lifespan data for Fig 5A–5E and S5 Fig. (DOCX)

S8 Table Statistical analysis of lifespan data for S7 Fig. (DOCX)

S9 Table Statistical analysis of lifespan data for S8 Fig. (DOCX)

S10 Table Statistical analysis of lifespan data for Fig 6. (DOCX)

S11 Table Statistical analysis of lifespan data for Fig 7 and S9 Fig. (DOCX)

S12 Table Statistical analysis of lifespan data for Fig 8A. (DOCX)

S13 Table Influence of *atg-18* mutations on the expression levels of ILP genes. (DOCX)
