# Supplementary figures and images for: The cell non-autonomous function of ATG-18 is essential for neuroendocrine regulation of Caenorhabditis elegans lifespan
Source: PLoS Genet. 2017 May 30;13(5):e1006764. doi: 10.1371/journal.pgen.1006764 (PMC5469504; doi:10.1371/journal.pgen.1006764)

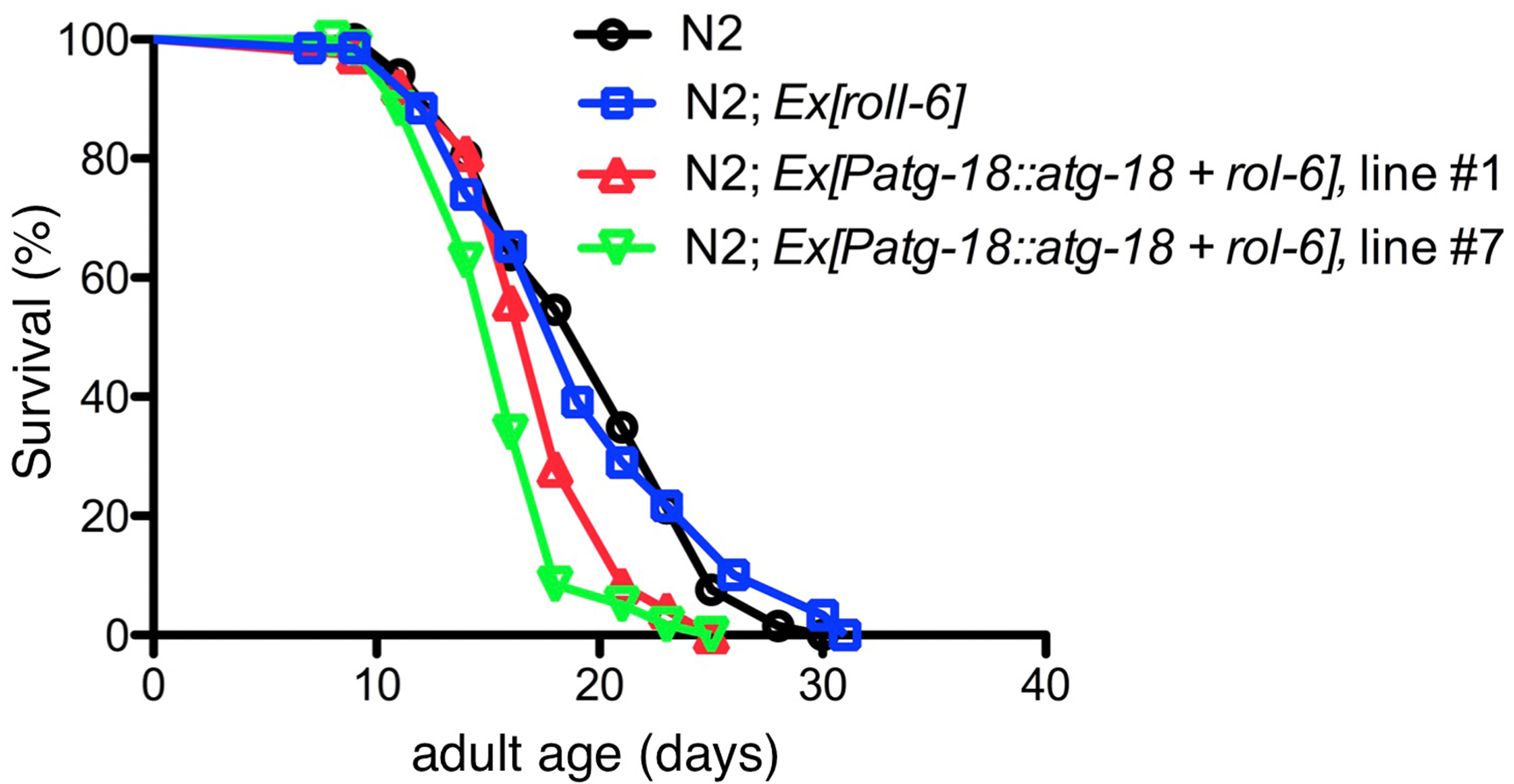

Supplement: S1 Fig — (TIF) [file pgen.1006764.s001.tif]

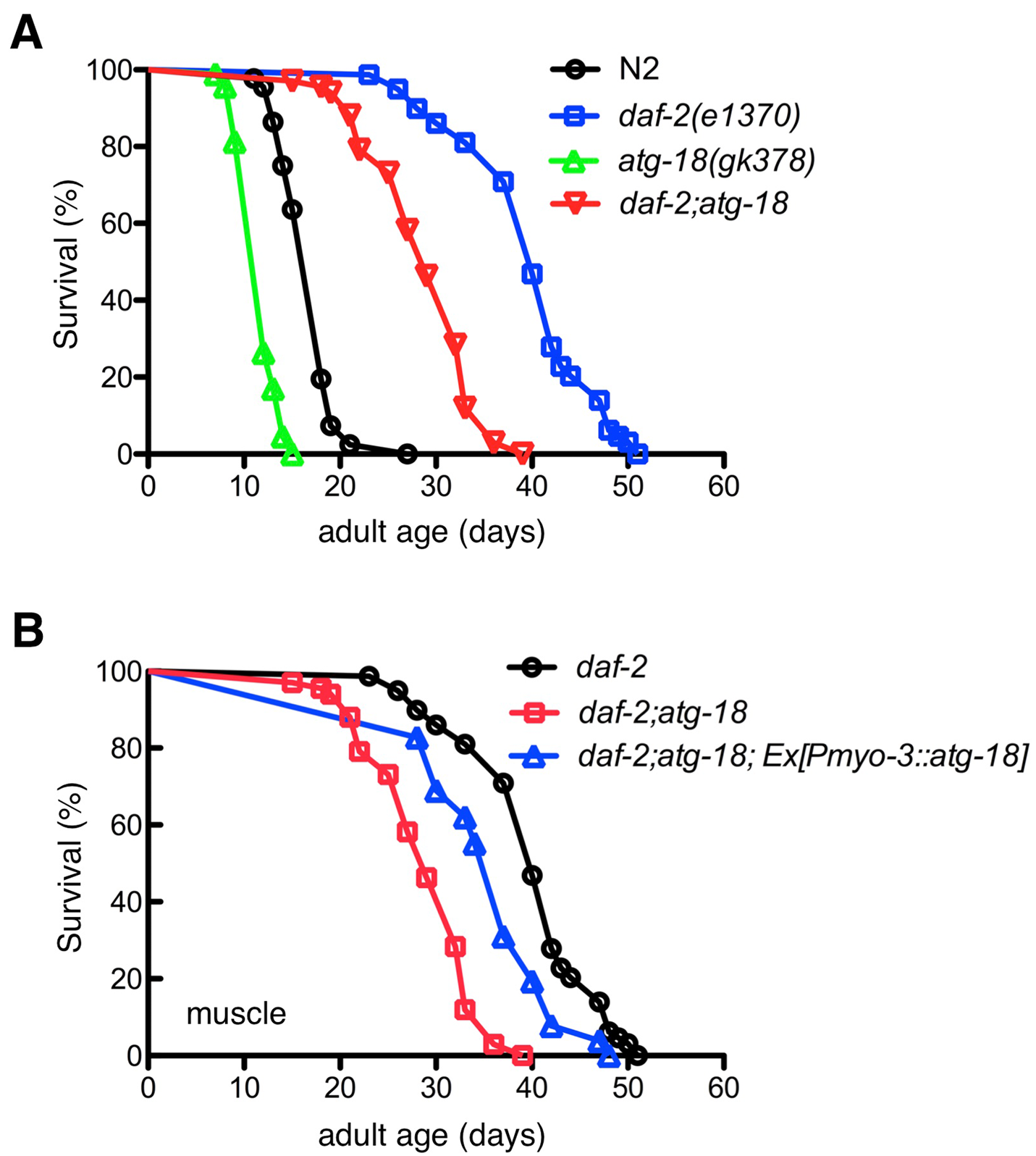

Supplement: S2 Fig — The atg-18(gk378) mutation significantly decreases the lifespan of daf-2(e1370) mutants (A) and muscle ATG-18 modestly increases the lifespan of daf-2(e1370);atg-18(gk378) mutants (B). (TIF) [file pgen.1006764.s002.tif]

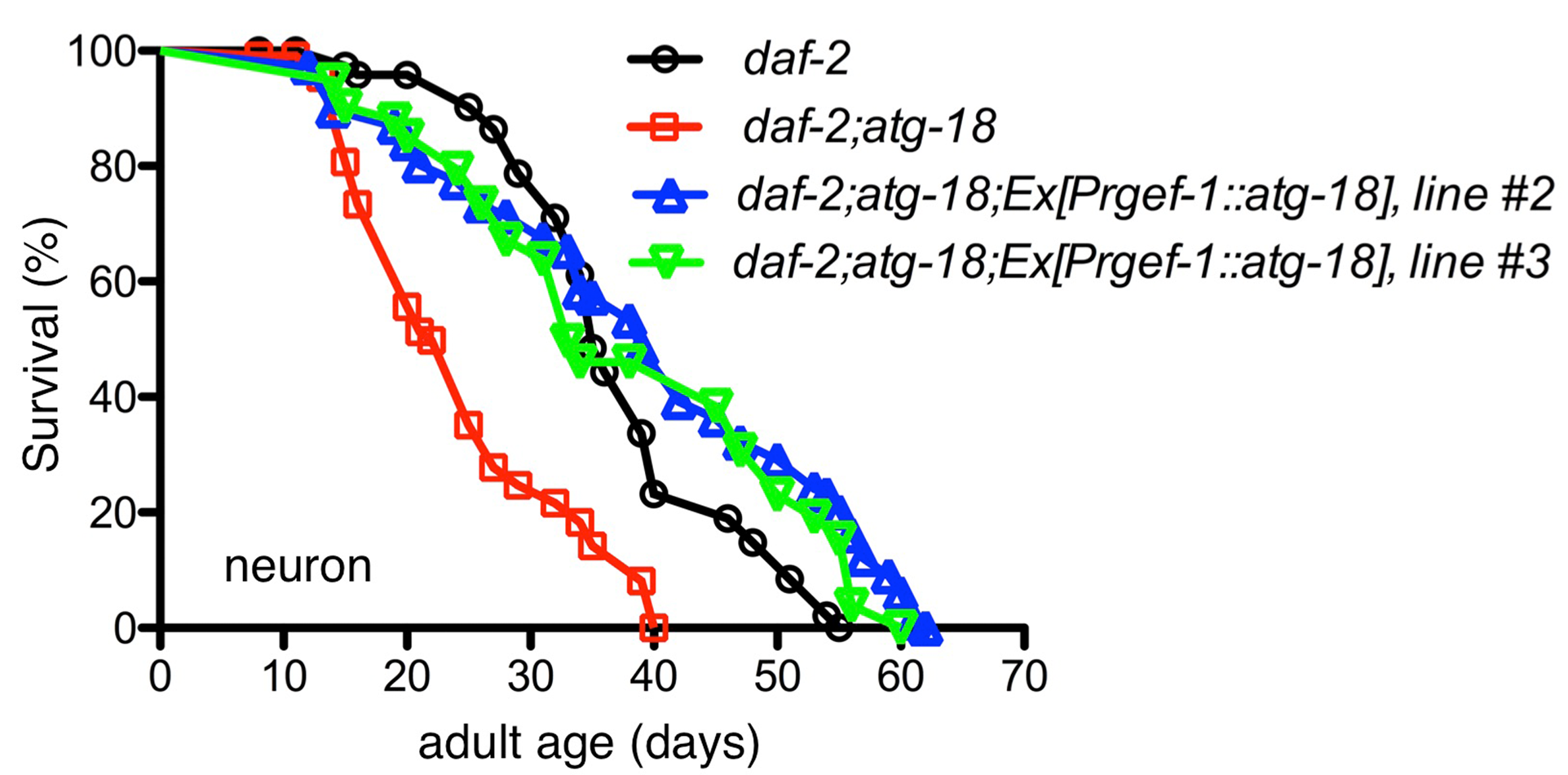

Supplement: S3 Fig — (TIF) [file pgen.1006764.s003.tif]

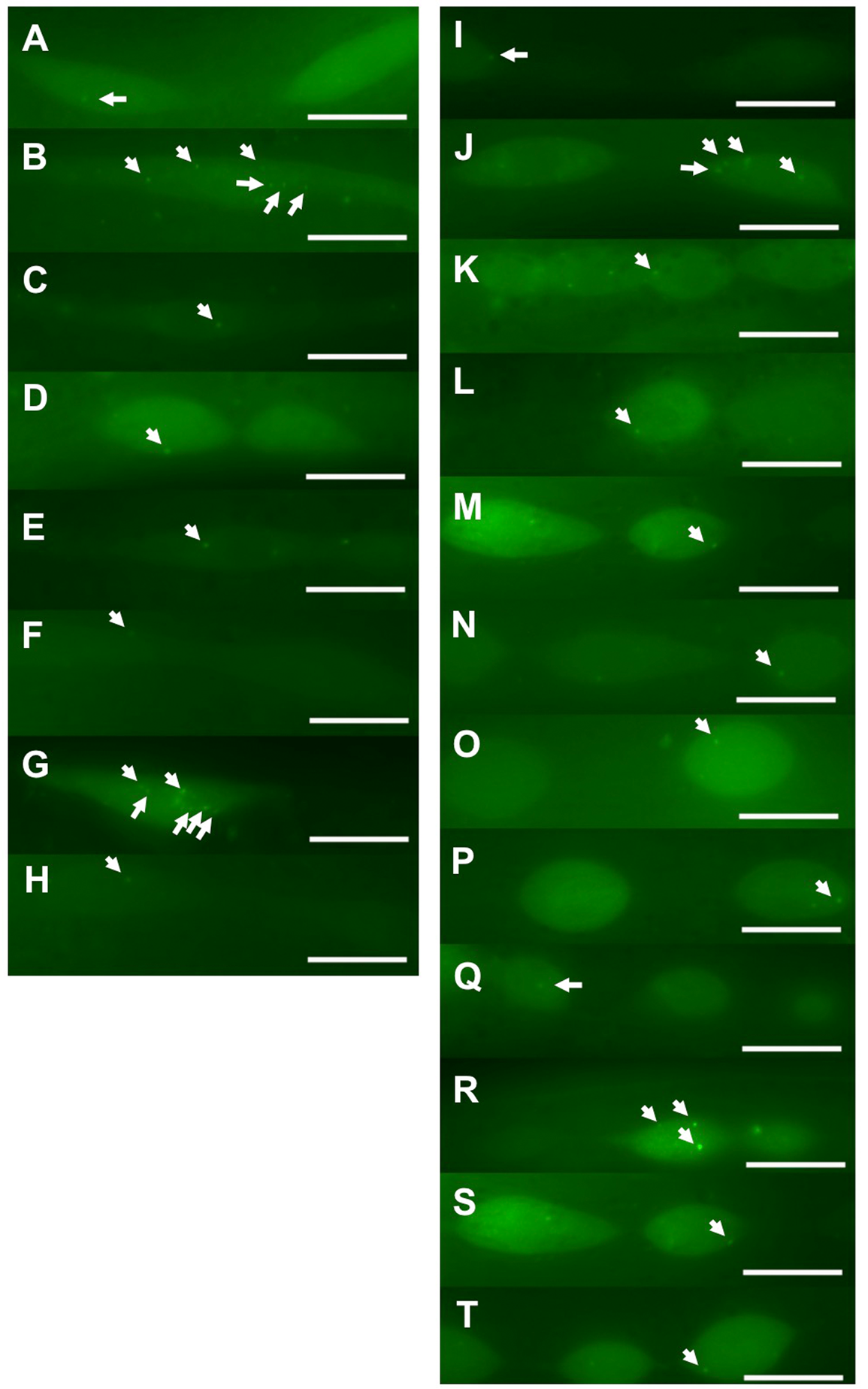

Supplement: S4 Fig — (A) N2 (B) daf-2(e1370) (C) atg-18(gk378) (D) daf-2;atg-18 (E) daf-2;atg-18; Ex[Punc-119::atg-18] (F) daf-2;atg-18; Ex[Pges-1::atg-18] (G) daf-2;atg-18; Ex[Pdpy-7::atg-18] (H) daf-2;atg-18; Ex[Pmyo-3::atg-18] (I) N2 +AL (J) N2 + DR (K) atg-18 + AL (L) atg-18 + DR (M) atg-18;Ex[Punc-119::atg-18] + AL (N) atg-18;Ex[Punc-119::atg-18] + DR (O) atg-18;Ex[Pges-1::atg-18] + AL (P) atg-18;Ex[Pges-1::atg-18] + DR (Q) atg-18;Ex[Pdpy-7::atg-18] + AL (R) atg-18;Ex[Pdpy-7::atg-18] + DR (S) atg-18;Ex[Pmyo-3::atg-18] + AL (T) atg-18;Ex[Pmyo-3::atg-18] + DR. Arrows denote representative autophagosomes. Scale bars: 10μM. (TIF) [file pgen.1006764.s004.tif]

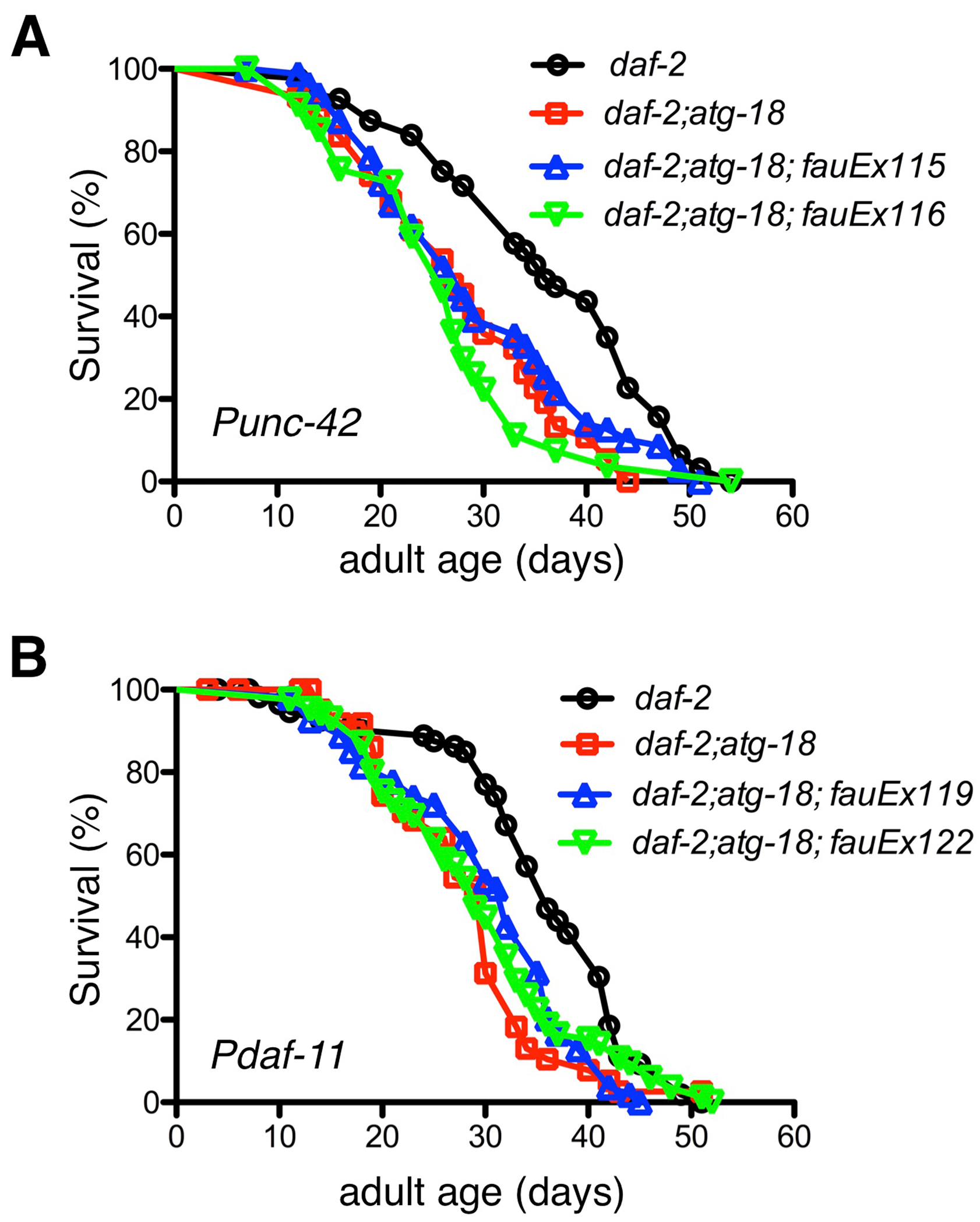

Supplement: S5 Fig — Expression of atg-18 in ASH neurons (Punc-42::atg-18) (A) and in ASE, ASI, ASJ, ASK, AWB and AWC chemosensory neurons (Pdaf-11::atg-18) (B) has no obvious effect on the lifespan daf-2(e1370);atg-18(gk378) mutants. (TIF) [file pgen.1006764.s005.tif]

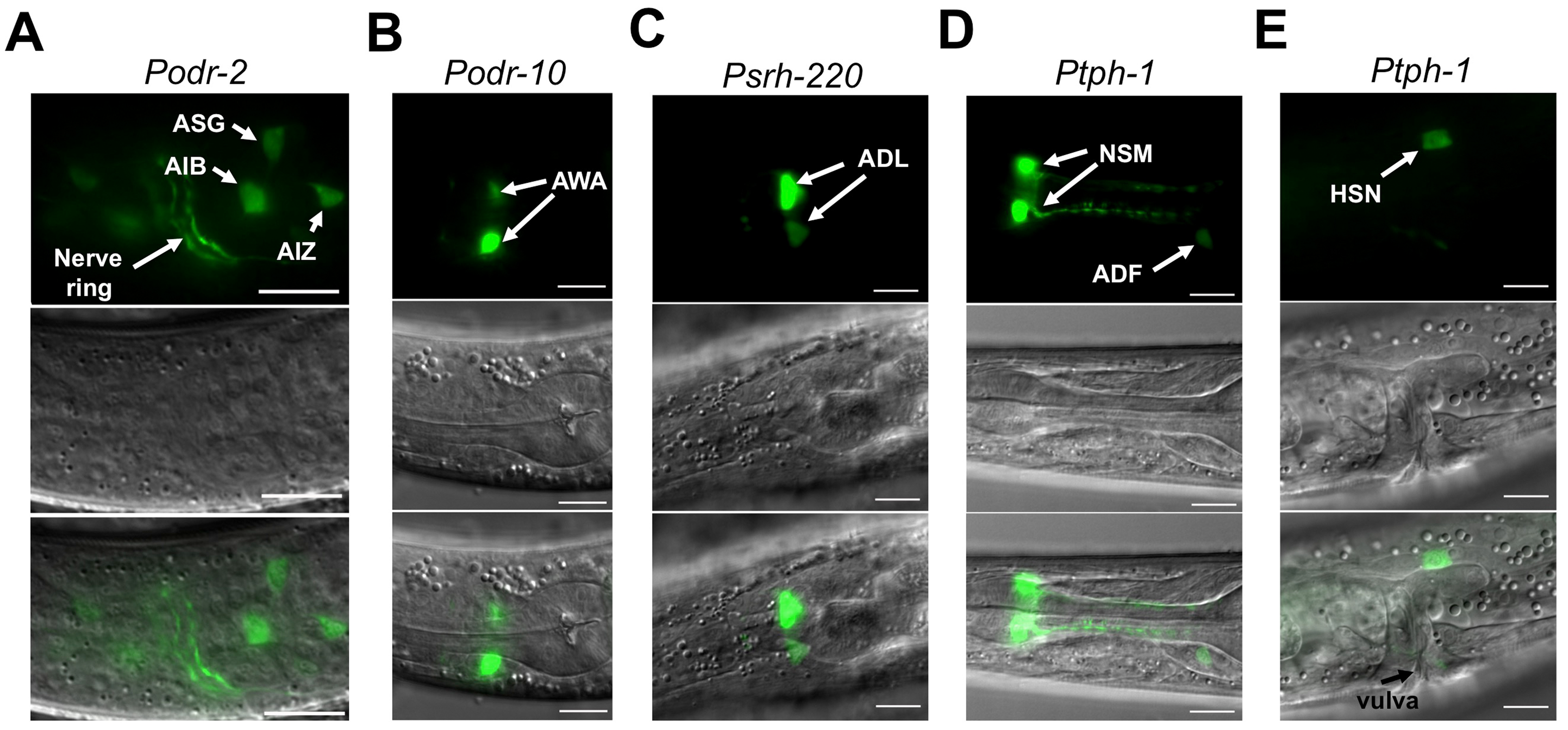

Supplement: S6 Fig — (A) The odr-2 promoter drives expression of the gfp::atg-18 reporter gene in ASG chemosensory neurons, the nerve ring and other neurons (see text for details). (B, C) The odr-10 and srh-220 promoters drive the expression of gfp::atg-18 in AWA and ADL chemosensory neurons, respectively. (D, E) Expression of gfp::atg-18 in ADF, NSM and HSN serotonergic neurons under the control of the tph-1 promoter. The anterior of the worm body in the image points to the left. Scale bars: 10μM. (TIF) [file pgen.1006764.s006.tif]

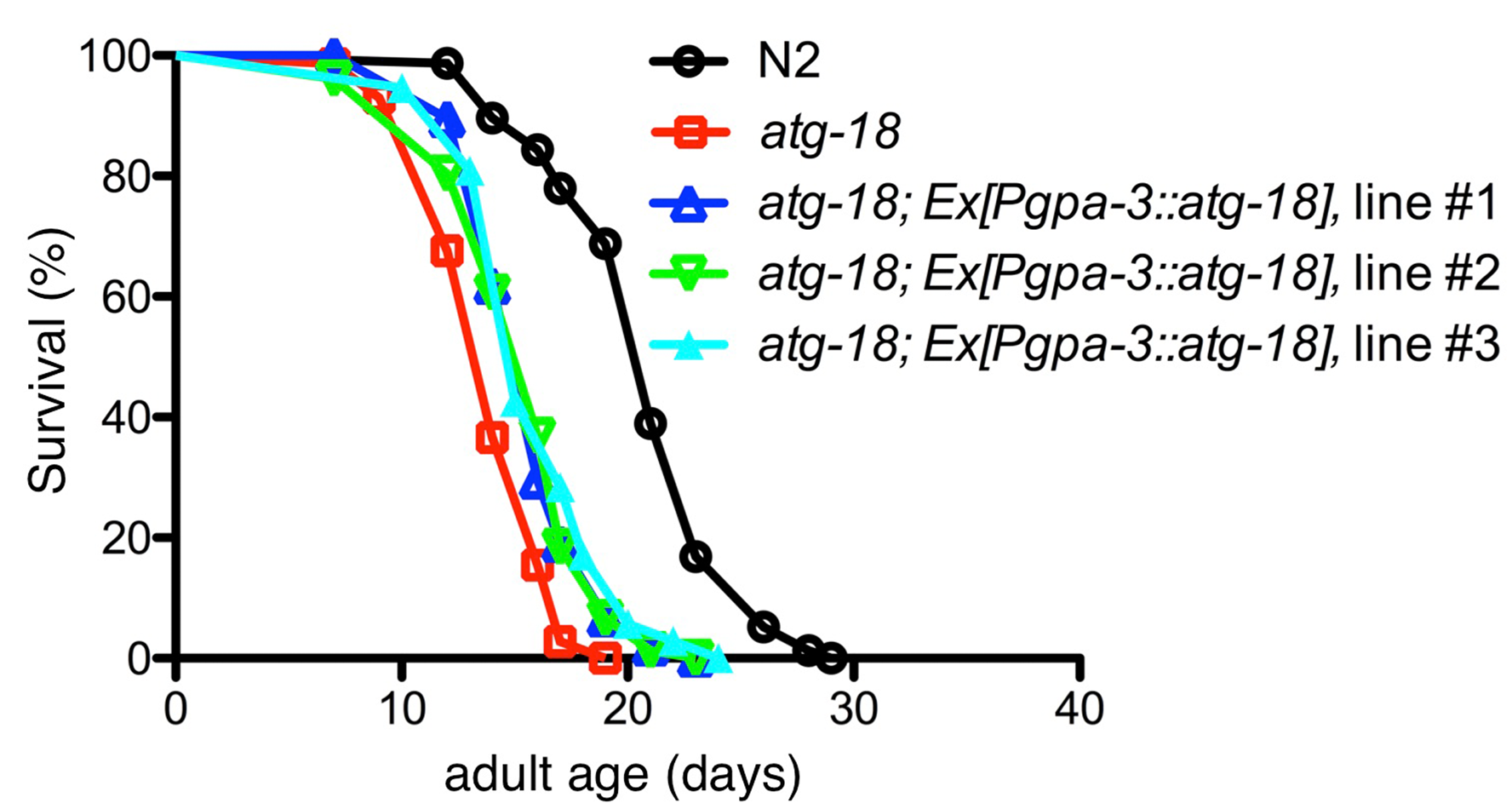

Supplement: S7 Fig — (TIF) [file pgen.1006764.s007.tif]

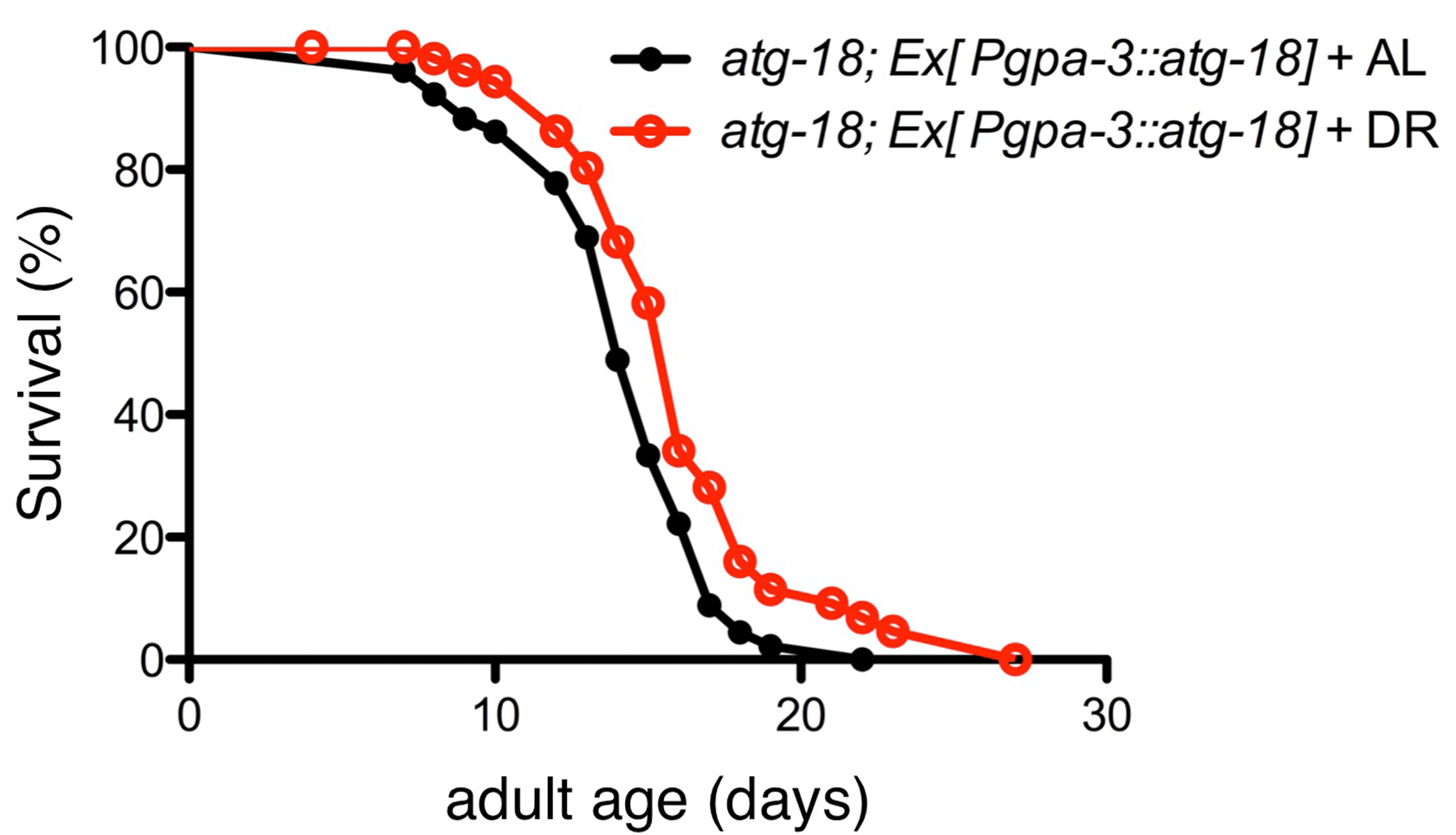

Supplement: S8 Fig — (TIF) [file pgen.1006764.s008.tif]

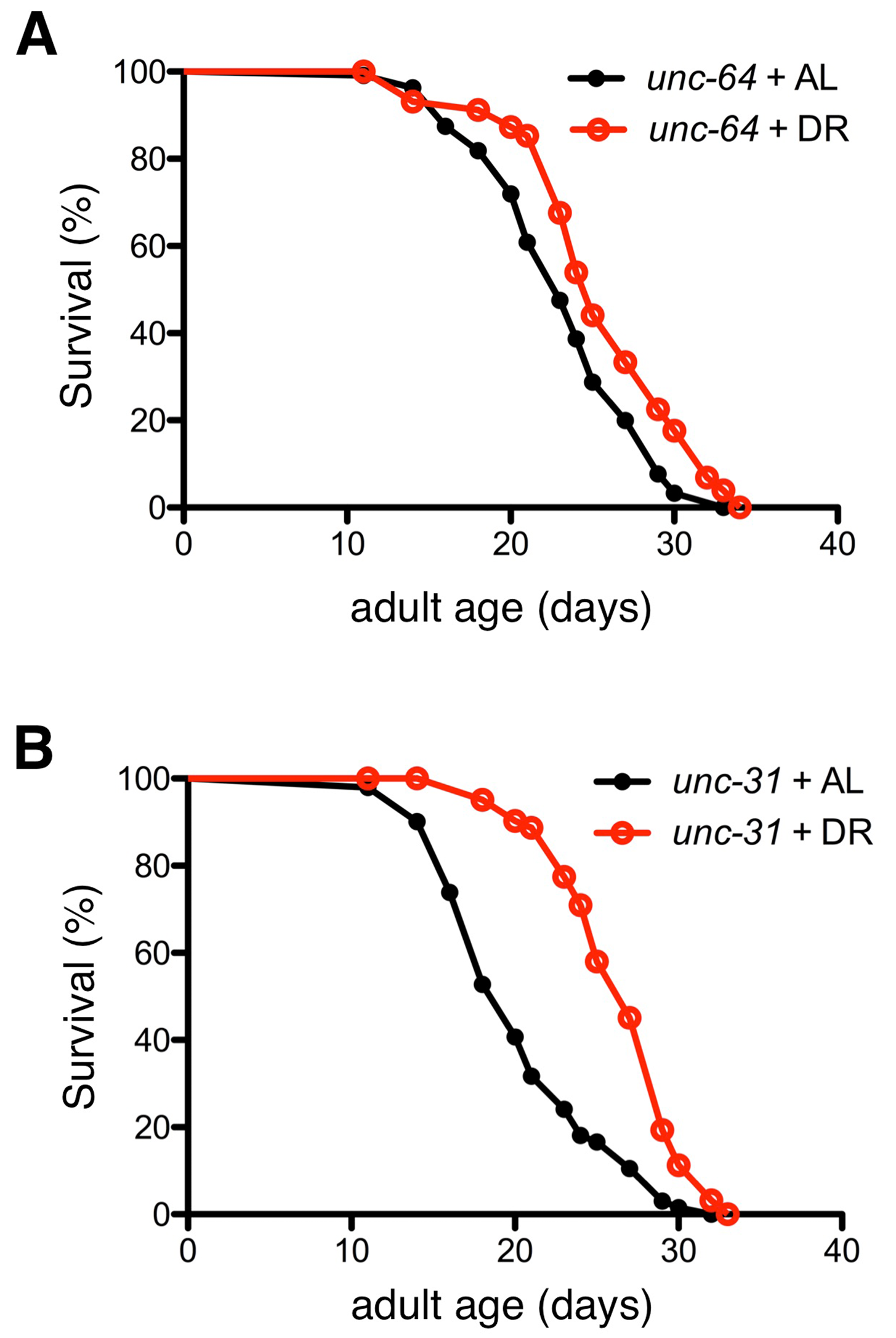

Supplement: S9 Fig — Dietary restriction extends the lifespan of unc-64 (A) and unc-31 (B) mutants. (TIF) [file pgen.1006764.s009.tif]

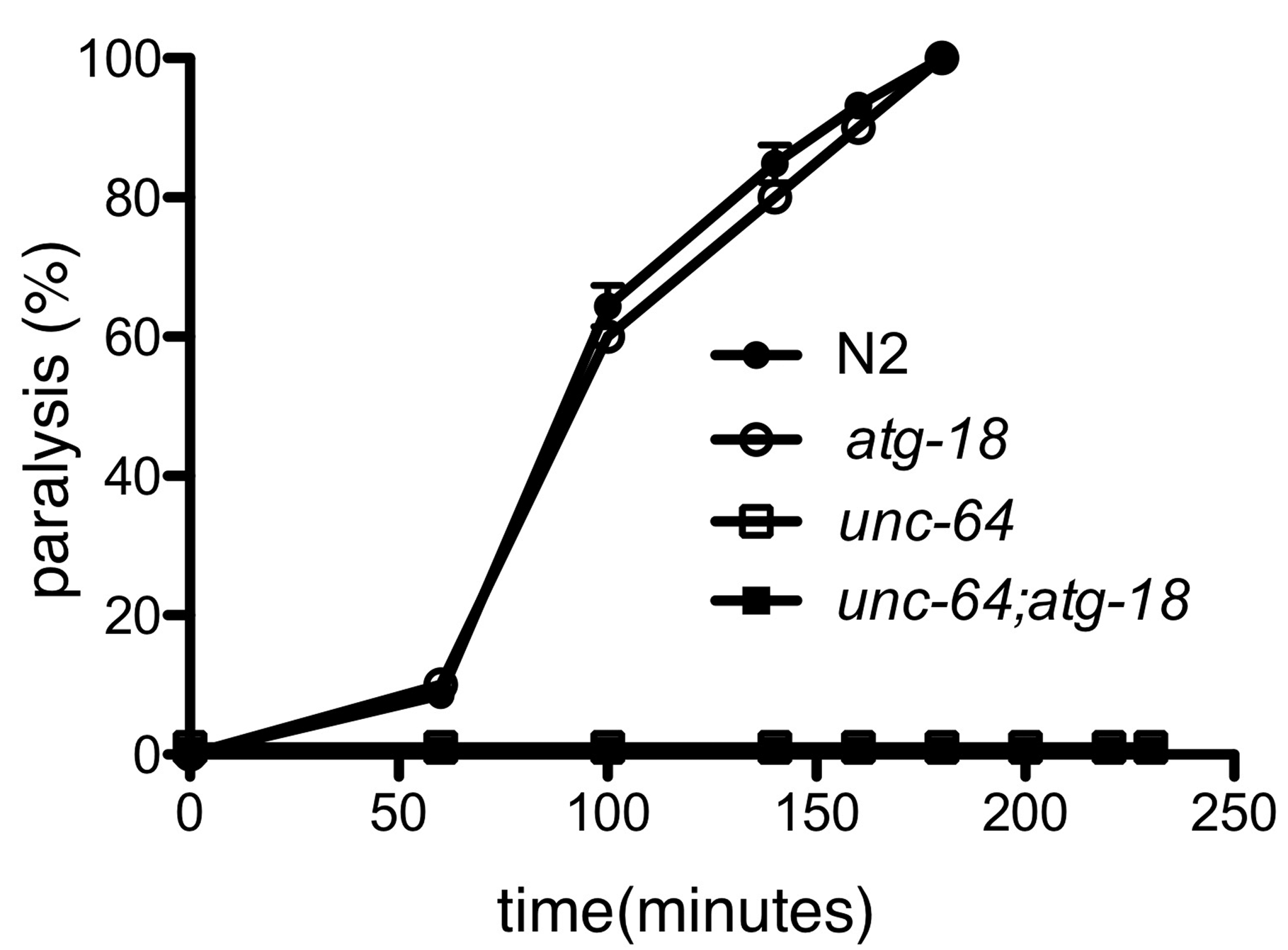

Supplement: S10 Fig — The paralysis was examined in triplicates for each sample at the following time points after drug treatment: 0 min, 100 min, 140 min, 160 min and 180 min. t-test was performed for statistical analysis at each time point. The p value at 100-minute time point is: 0.3210 for N2 v.s. atg-18, 0.5614 for unc-64 v.s. unc-64;atg-18 and <0.0001 for atg-18 v.s. unc-64;atg-18. Similar P values are obtained at each time point. (TIF) [file pgen.1006764.s010.tif]
