# Supplementary material for: The cell non-autonomous function of ATG-18 is essential for neuroendocrine regulation of Caenorhabditis elegans lifespan
Source: PLoS Genet. 2017 May 30;13(5):e1006764. doi: 10.1371/journal.pgen.1006764 (PMC5469504; doi:10.1371/journal.pgen.1006764)
Supplement: S1 Table — (DOCX) [file pgen.1006764.s011.docx]

**S1 Table. Statistical analysis of lifespan data for Fig 1**

| **Genotype** | **Lifespan (days)** | | **% of**  **control *^c^*** | **n *^d^***  **(censored)** | ***p* *^e^*** |
| --- | --- | --- | --- | --- | --- |
|  | **median *^a^*** | **max *^b^*** |  |  |  |
| N2  *atg-18* | 21,21,19  11,10,15 | 30,40,30  16,17,18 | /  52%,48%,79%*^f^* | 66(14),75(2),59(8)  60(3),76(1),75(7) | /  <0.0001, <0.0001, <0.0001 *^f^* |
| *atg-18; Ex[Patg-18::atg-18]* | 18,21,18 | 28,35,32 | 164%,210%,120%*^g^* | 75(10),53(20),65(4) | <0.0001,<0.0001, <0.0001 *^g^* |
| *atg-18; Ex[Punc-119::atg-18]* | 18,19,19 | 28,35,34 | 164%,190%,127% *^g^* | 70(0),54(6),75(7) | <0.0001,<0.0001, <0.0001 *^g^* |
| *atg-18; Ex[Pges-1::atg-18]* | 18,24,18 | 25,35,30 | 164%,240%,120% *^g^* | 56(0),59(27),67(6) | <0.0001,<0.0001, <0.0001 *^g^* |
| *atg-18; Ex[Pmyo-3::atg-18]*  ***atg-18;Ex[Pdpy-7::atg-18]* trial**  N2  *atg-18* | 16,17,15  24,21,19  13,11,15 | 21,24,21  36,29,30  17,16,18 | 145%,170%,100% *^g^*  /  54%,52%,79% *^f^* | 68(9),74(8),57(7)  62(1),66(0),59(8)  70(2),69(7),75(7) | <0.0001,<0.0001, <0.0001 *^g^*  /  <0.0001,<0.0001, <0.0001 *^f^* |
| *atg-18; Ex[Pdpy-7::atg-18]* | 14,14,15 | 23,22,19 | 108%,127%,100% *^g^* | 90(0),37(6),73(6) | 0.5312,0.0005,0.3420 *^g^* |

*^a^* Median lifespan for each trial

*^b^* Maximum lifespan for each trial

*^c^* Percentage of changes in median lifespan relative to corresponding control for each trial

*^d^* Numbers of animals counted for each trial (censored: animals died of internal hatching or lost during the experiments)

*^e^* *p* values (log-rank test) compared to corresponding control

*^f^* Compared to N2

*^g^* Compared to *atg-18*
