# Supplementary material for: The cell non-autonomous function of ATG-18 is essential for neuroendocrine regulation of Caenorhabditis elegans lifespan
Source: PLoS Genet. 2017 May 30;13(5):e1006764. doi: 10.1371/journal.pgen.1006764 (PMC5469504; doi:10.1371/journal.pgen.1006764)
Supplement: S2 Table — (DOCX) [file pgen.1006764.s012.docx]

**S2 Table. Statistical analysis of lifespan data for S1 Fig**

| **Genotype** | **Lifespan (days)** | | **% of control *^c^*** | **n *^d^***  **(censored)** | ***p* *^e^*** |
| --- | --- | --- | --- | --- | --- |
|  | **median *^a^*** | **max *^b^*** |  |  |  |
| N2 | 21,21 | 30,40 | / | 66(14),75(2) | / |
| N2 *+ rol-6* | 19,21 | 31,40 | 90%, 100% | 65(5),59(6) | 0.6166, 0.1771 |
| N2 + *Patg-18::atg-18 + rol-6, #1* | 18,17 | 25,38 | 86%,81% | 72(5),63(2) | 0.0005, <0.0001 |
| N2 + *Patg-18::atg-18 + rol-6, #7* | 16,14 | 25,24 | 76%,67% | 59(6),48(10) | <0.0001, <0.0001 |

*^a^* Median lifespan for each trial

*^b^* Maximum lifespan for each trial

*^c^* Percentage of changes in median lifespan relative to N2 for each trial

*^d^* Numbers of animals counted for each trial (censored: animals died of internal hatching or lost during the experiments)

*^e^p* values (log-rank test) compared to N2
