# Supplementary material for: The cell non-autonomous function of ATG-18 is essential for neuroendocrine regulation of Caenorhabditis elegans lifespan
Source: PLoS Genet. 2017 May 30;13(5):e1006764. doi: 10.1371/journal.pgen.1006764 (PMC5469504; doi:10.1371/journal.pgen.1006764)
Supplement: S3 Table — (DOCX) [file pgen.1006764.s013.docx]

**S3 Table. Statistical analysis of lifespan data for Fig 2**

| **Genotype** | **Lifespan (days)** | | **% of**  **control *^c^*** | **n *^d^***  **(censored)** | ***p* *^e^*** |
| --- | --- | --- | --- | --- | --- |
|  | **median *^a^*** | **max *^b^*** |  |  |  |
| N2 *+* AL  N2 *+* DR  *atg-18 +* AL  *atg-18 +* DR | 21,18  22,23  14,15  13, 15 | 22,34  35,32  16,16  16,16 | /  104%,127%  /  93%,100% | 68(9),32(7)  47(14),44(12)  98(7),44(16)  23(4),42(5) | /  <0.0001, <0.0001  /  0.0262,0.6007 |
| *atg-18; Ex[Punc-119::atg-18] +* AL  *atg-18; Ex[Punc-119::atg-18] +* DR  *atg-18; Ex[Pges-1::atg-18] +* AL  *atg-18; Ex[Pges-1::atg-18] +* DR  *atg-18; Ex[Pmyo-3::atg-18] +* AL  *atg-18; Ex[Pmyo-3::atg-18]* + DR | 21,23  21,26  21,21  21,23  15,17  16,17 | 25,28  34,31  22,28  35,30  21,25  23,27 | /  100%,113%  /  100%,110%  /  107%,100% | 65(10),30(4)  43(0),24(10)  73(13),39(13)  61(9),41(5)  38(8),44(3)  74(5),43(7) | /  0.0014,0.0009  /  <0.0001,0.0028  /  0.5440,0.0447 |
| *atg-18; Ex[Pdpy-7::atg-18] +* AL  *atg-18;Ex[Pdpy-7::atg-18] +* DR | 20,20  18,21 | 34,30  32,31 | /  90%,105% | 89(12),91(10)  62(10),101(7) | /  0.2112,0.8799 |

*^a^* Median lifespan for each trial

*^b^* Maximum lifespan for each trial

*^c^* Percentage of changes in median lifespan (DR) relative to corresponding control (AL) for each trial

*^d^* Numbers of animals counted for each trial (censored: animals died of internal hatching or lost during the experiments)

*^e^* *p* values (log-rank test) compared to corresponding control (DR vs. AL)
