# Supplementary material for: The cell non-autonomous function of ATG-18 is essential for neuroendocrine regulation of Caenorhabditis elegans lifespan
Source: PLoS Genet. 2017 May 30;13(5):e1006764. doi: 10.1371/journal.pgen.1006764 (PMC5469504; doi:10.1371/journal.pgen.1006764)
Supplement: S4 Table — (DOCX) [file pgen.1006764.s014.docx]

**S4 Table. Statistical analysis of lifespan data for Fig 3 and S2 Fig**

| **Genotype** | **Lifespan (days)** | | **% of**  **control *^c^*** | **n *^d^***  **(censored)** | ***p* *^e^*** |
| --- | --- | --- | --- | --- | --- |
|  | **median *^a^*** | **max *^b^*** |  |  |  |
| N2  *atg-18*  *daf-2*  *daf-2;atg-18* | 24,18,19  12,12,13  36,32,35  22,24,25 | 37,27,28  15,14,19  51,46,52  39,35,42 | /  50%,67%,68%  150%,178%,184  61%,75%,71% | 73(1),42(2),77(2)  65(4),84(7),77(4)  78(0),74(16),58(25)  80(0),81(7),76(17) | /  <0.0001,<0.0001, <0.0001 *^f^*  <0.0001,<0.0001, <0.0001 *^f^*  <0.0001,<0.0001, <0.0001 *^g^* |
| *daf-2;atg-18; Ex[Patg-18::atg-18]* | 35,35,36 | 52,50,47 | 159%,146%,144% | 75(1),84(12),74(5) | <0.0001,<0.0001, <0.0001 *^h^* |
| *daf-2;atg-18; Ex[Punc-119::atg-18]*  *daf-2;atg-18; Ex[Pges-1::atg-18]*  ***daf-2;atg-18; Ex[Pmyo-3::atg-18]***  *daf-2*  *daf-2;atg-18*  *daf-2;atg-18; Ex[Pmyo-3::atg-18]*  ***daf-2;atg-18; Ex[Pdpy-7::atg-18]***  *daf-2*  *daf-2;atg-18*  *daf-2;atg-18; Ex[Pdpy-7::atg-18]* | 43,30,40  32,35,35  40, 32,35  29, 24,25  37, 26,29  35,37,26  27,26,19  36,35,23 | 55,49,55  48,56,53    51, 46,52  39, 35,42  48, 41,47  51,43,39  48,40,28  57,45,42 | 195%,125%,160%  145%,146%,140%  138%,133%,140%  /  128%,108%,116%  130%,142%,137%  /  133%,135%,121% | 83(0),73(14),84(3)  76(6),61(1),111(5)  77(2), 74(16),58(25)  67(0), 81(7),76(17)  28(1), 72(11),78(9)  60(28),77(0),76(6)  59(21),93(2),91(10)  64(7),68(3),97(14) | <0.0001,<0.0001, <0.0001 *^h^*  <0.0001,<0.0001, <0.0001 *^h^*  <0.0001,<0.0001, <0.0001 *^f^*  /  <0.0001,<0.0001,0.0108 *^h^*  <0.0001,<0.0001,<0.0001 *^h^*  /  <0.0001,<0.0001,<0.0001 *^h^* |

*^a^* Median lifespan for each trial

*^b^* Maximum lifespan for each trial

*^c^* Percentage of changes in median lifespan relative to corresponding control for each trial

*^d^* Numbers of animals counted for each trial (censored: animals died of internal hatching or lost during the experiments)

*^e^* *p* values (log-rank test) compared to corresponding control

*^f^* Compared to N2

*^g^* Compared to *daf-2*

*^h^* Compared to *daf-2;atg-18*
