# Supplementary material for: The cell non-autonomous function of ATG-18 is essential for neuroendocrine regulation of Caenorhabditis elegans lifespan
Source: PLoS Genet. 2017 May 30;13(5):e1006764. doi: 10.1371/journal.pgen.1006764 (PMC5469504; doi:10.1371/journal.pgen.1006764)
Supplement: S5 Table — (DOCX) [file pgen.1006764.s015.docx]

**S5 Table. Statistical analysis of lifespan data for S3 Fig**

| **Genotype** | **Lifespan (days)** | | **% of control *^c^*** | **n *^d^***  **(censored)** | ***p* *^e^*** |
| --- | --- | --- | --- | --- | --- |
|  | **median *^a^*** | **max *^b^*** |  |  |  |
| *daf-2* | 35,35 | 55,52 | 159,130 | 50(51),58(25) | <0.0001,<0.0001 |
| *daf-2;atg-18* | 22,27 | 40,37 | / | 74(46),76(13) | / |
| *daf-2;atg-18;Ex[Prgef-1::atg-18] #2* | 39,32 | 62,57 | 177,119 | 101(6),92(1) | <0.0001, <0.0001 |
| *daf-2;atg-18;Ex[Prgef-1::atg-18] #3* | 33,41 | 60,57 | 150,152 | 44(52),96(0) | <0.0001, <0.0001 |

*^a^* Median lifespan for each trial

*^b^* Maximum lifespan for each trial

*^c^* Percentage of changes in median lifespan relative to *daf-2;atg-18* for each trial

*^d^* Numbers of animals counted for each trial (censored: animals died of internal hatching or lost during the experiments)

*^e^* *p* values (log-rank test) compared to corresponding control *daf-2;atg-18*
