# Supplementary material for: The cell non-autonomous function of ATG-18 is essential for neuroendocrine regulation of Caenorhabditis elegans lifespan
Source: PLoS Genet. 2017 May 30;13(5):e1006764. doi: 10.1371/journal.pgen.1006764 (PMC5469504; doi:10.1371/journal.pgen.1006764)
Supplement: S6 Table — (DOCX) [file pgen.1006764.s016.docx]

**S6 Table. Statistical analysis of autophagy induction**

| **Genotype** | **Autophagosomes per seam cell** | | **n *^a^*** | ***p*** |
| --- | --- | --- | --- | --- |
|  | **mean** | **Standard error of mean** |  |  |
| N2  *daf-2* | 0.9831  5.2344 | 0.104  0.1633 | 59  64 | /  <0.0001*^b^* |
| *daf-2;atg-18* | 1.2794 | 0.0933 | 68 | <0.0001*^c^* |
| *daf-2;atg-18; Ex[Punc-119::atg-18]* | 1.069 | 0.0709 | 87 | <0.0001 *^c^* |
| *daf-2;atg-18; Ex[Pges-1::atg-18]* | 1.04 | 0.0746 | 75 | <0.0001 *^c^* |
| *daf-2;atg-18; Ex[Pdpy-7::atg-18]* | 4.9155 | 0.1711 | 71 | 0.1821 *^c^* |
| *daf-2;atg-18; Ex[Pmyo-3::atg-18]* | 0.6392 | 0.0642 | 97 | <0.0001 *^c^* |
| N2 + AL  N2 + DR  *atg-18* + AL  *atg-18* + DR | 0.3833  2.8876  0.6433  0.7671 | 0.0633  0.1709  0.0723  0.0817 | 60  89  82  73 | /  <0.0001*^d^*  /  0.2681*^d^* |
| *atg-18; Ex[Punc-119::atg-18]* + AL | 0.7463 | 0.0911 | 67 | / |
| *atg-18; Ex[Punc-119::atg-18]* + DR  *atg-18; Ex[Pges-1::atg-18]* + AL  *atg-18; Ex[Pges-1::atg-18]* + DR  *atg-18; Ex[Pdpy-7::atg-18]* + AL  *atg-18; Ex[Pdpy-7::atg-18]* + DR | 0.6579  0.8442  0.7447  0.6235  2.8056 | 0.087  0.0786  0.0708  0.0689  0.1366 | 76  77  94  85  72 | 0.4845*^d^*  /  0.348 *^d^*  /  <0.0001 *^d^* |
| *atg-18; Ex[Pmyo-3::atg-18]* + AL  *atg-18; Ex[Pmyo-3::atg-18]* + DR | 0.5904  0.6196 | 0.0647  0.0671 | 83  92 | /  0.7555*^d^* |

*^a^* Total number of seam cells counted for autophagy induction. At least twenty L3 or L4 stage animals were examined in each group

*^b^* *p* values (t-test) compared to N2

*^c^ p* values (t-test) compared to *daf-2* mutants

*^d^* *p* values (t-test) compared to control animals fed *ad libitum* (AL)
