# Supplementary material for: The cell non-autonomous function of ATG-18 is essential for neuroendocrine regulation of Caenorhabditis elegans lifespan
Source: PLoS Genet. 2017 May 30;13(5):e1006764. doi: 10.1371/journal.pgen.1006764 (PMC5469504; doi:10.1371/journal.pgen.1006764)
Supplement: S7 Table — (DOCX) [file pgen.1006764.s017.docx]

**S7 Table. Statistical analysis of lifespan data for Fig 5 A-E and S5 Fig**

| **Genotype** | **Lifespan (days)** | | **% of control *^c^*** | **n *^d^***  **(censored)** | ***p* *^e^*** |
| --- | --- | --- | --- | --- | --- |
|  | **median *^a^*** | **max *^b^*** |  |  |  |
| ***Pgpa-3::atg-18 rescue***  *daf-2* | 36,33 | 54,53 | 171%,132% | 54(32),53(25) | <0.0001,<0.0001 |
| *daf-2;atg-18* | 21,25 | 35,36 | / | 101(12),48(33) | / |
| *daf-2;atg-18;fauEx88* | 41,36 | 63,57 | 195%,144% | 82(7),69(12) | <0.0001,<0.0001 |
| *daf-2;atg-18;fauEx89* | 30,39 | 61,56 | 143%,156% | 37(16),64(6) | <0.0001,<0.0001 |
| *daf-2;atg-18;fauEx90* | 40,34 | 64,53 | 190%,136% | 92(15),64(13) | <0.0001,<0.0001 |
| *daf-2;atg-18;fauEx91* | 41,41 | 63,58 | 195%,164% | 69(13),70(14) | <0.0001,<0.0001 |
| ***Podr-2::atg-18 rescue***  *daf-2*  *daf-2;atg-18*  *daf-2;atg-18;fauEx101* | 45,42  25,28  40,35 | 57,51  36,46  57,55 | 180%,150%  /  160%,125% | 37(61),94(1)  56(24),50(28)  81(14),73(21) | <0.0001,<0.0001  /  <0.0001,<0.0001 |
| *daf-2;atg-18;fauEx102* | 40,40 | 57,57 | 160%,143% | 57(16),51(20) | <0.0001,<0.0001 |
| ***Podr-10::atg-18 rescue***  *daf-2*  *daf-2;atg-18*  *daf-2;atg-18;fauEx82* | 35,32  23,18  33,24 | 50,47  30,32  51,35 | 152%,178%  /  143%,133% | 75(20),30(47)  48(41),53(4)  40(25),69(20) | <0.0001,<0.0001  /  <0.0001,<0.0001 |
| *daf-2;atg-18;fauEx83* | 33,21 | 51,30 | 143%,117% | 35(20),35(6) | <0.0001,0.0019 |
| ***Ptph-1::atg-18 rescue***  *daf-2*  *daf-2;atg-18*  *daf-2;atg-18;fauEx86* | 45,42  25,28  31,36 | 57,51  36,46  51,50 | 180%,150%  /  124%,129% | 37(61),94(1)  56(24),50(28)  24(14),65(41) | <0.0001,<0.0001  /  <0.0001,0.0012 |
| *daf-2;atg-18;fauEx87* | 35,38 | 50,56 | 140%,136% | 17(12),44(10) | 0.0004,0.0011 |
| ***Psrh-220::atg-18 rescue***  *daf-2*  *daf-2;atg-18*  *daf-2;atg-18;fauEx124* | 37,32  17,18  37,23 | 51,47  29,32  46,36 | 218%,178%  /  218%,128% | 73(19),30(47)  45(35),53(4)  51(26),66(15) | <0.0001, <0.0001  /  <0.0001, <0.0001 |
| *daf-2;atg-18;fauEx85*  ***Punc-42::atg-18 rescue***  *daf-2*  *daf-2;atg-18*  *daf-2;atg-18;fauEx115*  *daf-2;atg-18;fauEx116*  ***Pdaf-11::atg-18 rescue***  *daf-2*  *daf-2;atg-18*  *daf-2;atg-18;fauEx119*  *daf-2;atg-18;fauEx122* | 33,21  36,38  27,28  27,24  26,27  36,37  30,26  32,28  29,25 | 46,33  54,57  44,48  51,51  54,52  49,53  43,40  45,52  43,41 | 194%,117%  133%,136%  /  100%,86%  96%,96%  120%,142%  /  107%,108%  97%,96% | 30(20),44(9)  54(32),43(0)  81(7),69(0)  78(4),60(0)  30(5),66(0)  70(118),77(0)  43(37),93(2)  54(0),83(11)  85(0),25(2) | <0.0001, 0.0001  <0.0001,<0.0001  /  0.1816,0.8716  0.3174,0.1367  <0.0001,<0.0001  /  0.1865,0.0100  0.5324,0.3300 |

*^a^* Median lifespan for each trial

*^b^* Maximum lifespan for each trial

*^c^* Percentage of changes in median lifespan relative to *daf-2;atg-18* for each trial

*^d^* Numbers of animals counted for each trial (censored: animals died of internal hatching or lost during the experiments)

*^e^* *p* values (log-rank test) compared to *daf-2;atg-18*
