# Supplementary material for: The cell non-autonomous function of ATG-18 is essential for neuroendocrine regulation of Caenorhabditis elegans lifespan
Source: PLoS Genet. 2017 May 30;13(5):e1006764. doi: 10.1371/journal.pgen.1006764 (PMC5469504; doi:10.1371/journal.pgen.1006764)
Supplement: S8 Table — (DOCX) [file pgen.1006764.s018.docx]

**S8 Table. Statistical analysis of lifespan data for S7 Fig**

| **Genotype** | **Lifespan (days)** | | **% of control *^c^*** | **n *^d^***  **(censored)** | ***p* *^e^*** |
| --- | --- | --- | --- | --- | --- |
|  | **median *^a^*** | **max *^b^*** |  |  |  |
| N2 | 21,19 | 29,24 | 150%,136% | 77(0),55(21) | <0.0001,<0.0001 |
| *atg-18* | 14,14 | 19,16 | / | 71(0),77(12) | / |
| *atg-18* + *Pgpa-3::atg-18, #1* | 16,14 | 23,16 | 114%,100% | 105(1),63(18) | <0.0001, 0.3435 |
| *atg-18* + *Pgpa-3::atg-18, #2*  *atg-18* + *Pgpa-3::atg-18, #3* | 16,14  15,12 | 23,17  24,16 | 114%,100%  107%,86% | 76(2),74(16)  71(3),75(0) | 0.0002, 0.1209  <0.0001, 0.0011 |

*^a^* Median lifespan for each trial

*^b^* Maximum lifespan for each trial

*^c^* Percentage of changes in median lifespan relative to *atg-18* for each trial

*^d^* Numbers of animals counted for each trial (censored: animals died of internal hatching or lost during the experiments)

*^e^* *p* values (log-rank test) compared to *atg-18*
