# Supplementary material for: The cell non-autonomous function of ATG-18 is essential for neuroendocrine regulation of Caenorhabditis elegans lifespan
Source: PLoS Genet. 2017 May 30;13(5):e1006764. doi: 10.1371/journal.pgen.1006764 (PMC5469504; doi:10.1371/journal.pgen.1006764)
Supplement: S9 Table — (DOCX) [file pgen.1006764.s019.docx]

**S9 Table. Statistical analysis of lifespan data for S8 Fig**

| **Genotype** | **Lifespan (days)** | | **% of**  **control *^c^*** | **n *^d^***  **(censored)** | ***p* *^e^*** |
| --- | --- | --- | --- | --- | --- |
|  | **median *^a^*** | **max *^b^*** |  |  |  |
| *atg-18; Ex[Pgpa-3::atg-18] +* AL  *atg-18; Ex[Pgpa-3::atg-18] +* DR | 14,14  16,15 | 22,19  27,30 | /  114%,107% | 46(6),56(14)  49(6),50(16) | /  0.0070,0.0454 |

*^a^* Median lifespan for each trial

*^b^* Maximum lifespan for each trial

*^c^* Percentage of changes in median lifespan (DR) relative to corresponding control (AL) for each trial

*^d^* Numbers of animals counted for each trial (censored: animals died of internal hatching or lost during the experiments)

*^e^* *p* values (log-rank test) compared to corresponding control (DR vs. AL)
