# Supplementary material for: The cell non-autonomous function of ATG-18 is essential for neuroendocrine regulation of Caenorhabditis elegans lifespan
Source: PLoS Genet. 2017 May 30;13(5):e1006764. doi: 10.1371/journal.pgen.1006764 (PMC5469504; doi:10.1371/journal.pgen.1006764)
Supplement: S10 Table — (DOCX) [file pgen.1006764.s020.docx]

**S10 Table. Statistical analysis of lifespan data for Fig 6**

| **Genotype** | **Lifespan (days)** | | **% of control *^c^*** | **n *^d^***  **(censored)** | ***p* *^e^*** |
| --- | --- | --- | --- | --- | --- |
|  | **median *^a^*** | **max *^b^*** |  |  |  |
| *unc-64* | 18,18 | 28,27 | 129%,129% | 87(0),59(8) | <0.0001,<0.0001 |
| *unc-64;atg-18* | 14,14 | 18,22 | / | 74(4),93(4) | / |
| *unc-64;atg-18;fauEx48* | 16,14 | 21,27 | 114%,100% | 54(22),83(0) | <0.0001, 0.1148 |
| *unc-64;atg-18;fauEx49* | 16,15 | 21,35 | 114%,107% | 79(5),81(0) | <0.0001, 0.1848 |
| *unc-64;atg-18;fauEx69* | 21,20 | 28,29 | 150%,143% | 64(2),71(13) | <0.0001, <0.0001 |
| *unc-64;atg-18;fauEx70* | 16,17 | 28,24 | 114%,121% | 50(1),64(3) | 0.0005, <0.0001 |
| *unc-31* | 24,26 | 45,42 | 141%,137% | 74(3),82(0) | <0.0001,<0.0001 |
| *unc-31;atg-18* | 17,19 | 24,34 | / | 50(7),58(2) | / |
| *unc-31;atg-18;fauEx156* | 20,21 | 36,41 | 118%,111% | 75(7),79(1) | 0.0004,0.0027 |
| *unc-31;atg-18;fauEx160* | 20,26 | 45,40 | 118%,137% | 48(5),59(1) | 0.0300, <0.0001 |
| *unc-31;atg-18;fauEx157* | 17,16 | 27,36 | 100%,84% | 38(9),72(2) | 0.6034,0.6974 |
| *unc-31;atg-18;fauEx158* | 16,17 | 28,34 | 94%,89% | 65(4),72(2) | 0.3698,0.4785 |
|  |  |  |  |  |  |
| *daf-2unc64* | 42,36 | 53,53 | 300%,157% | 57(15),46(27) | <0.0001,<0.0001 |
| *daf-2unc64;atg-18* | 14,23 | 23,40 | / | 89(14),84(7) | / |
| *daf-2unc-64;atg-18;fauEx181* | 23,34 | 51,53 | 164%,148% | 80(1),74(1) | <0.0001, 0.0001 |
| *daf-2unc-64;atg-18;fauEx216* | 30,34 | 51,53 | 214%,148% | 102(4),98(1) | <0.0001, 0.0001 |
|  |  |  |  |  |  |
| *daf-2unc-64* | 39,40 | 68,69 | 170%,114% | 82(5),75(7) | <0.0001,<0.0001 |
| *daf-2unc-64;atg-18* | 23,35 | 46,53 | / | 79(0),108(0) | / |
| *daf-2unc-64;atg-18;fauEx182* | 35,39 | 62,64 | 152%,111% | 90(7),58(2) | <0.0001,<0.0001 |
| *daf-2unc-64;atg-18;fauEx186* | 35,30 | 59,52 | 152%,86% | 52(8),57(2) | <0.0001,<0.0001 |

*^a^* Median lifespan for each trial

*^b^* Maximum lifespan for each trial

*^c^* Percentage of changes in median lifespan relative to corresponding control (*unc-64;atg-18* or *unc-31;atg-18*) for each trial

*^d^* Numbers of animals counted for each trial (censored: animals died of internal hatching or lost during the experiments)

*^e^* *p* values (log-rank test) compared to corresponding control (*unc-64;atg-18*, *unc-31;atg-18* or *daf-2unc-64;atg-18*)
