# Supplementary material for: The cell non-autonomous function of ATG-18 is essential for neuroendocrine regulation of Caenorhabditis elegans lifespan
Source: PLoS Genet. 2017 May 30;13(5):e1006764. doi: 10.1371/journal.pgen.1006764 (PMC5469504; doi:10.1371/journal.pgen.1006764)
Supplement: S11 Table — (DOCX) [file pgen.1006764.s021.docx]

**S11 Table. Statistical analysis of lifespan data for Fig 7 and S9 Fig**

| **Genotype** | **Lifespan (days)** | | **% of**  **control *^c^*** | **n *^d^***  **(censored)** | ***p* *^e^*** |
| --- | --- | --- | --- | --- | --- |
|  | **median *^a^*** | **max *^b^*** |  |  |  |
| N2 *+* AL  N2 *+* DR  *unc-64 +* AL  *unc-64 +* DR  *unc-64;atg-18 +* AL  *unc-64;atg-18 +* DR  *unc-64;atg-18;Ex[Punc-119::atg-18] +* AL  *unc-64;atg-18;Ex[Punc-119::atg-18] +* DR  *unc-64;atg-18;Ex[Pges-1::atg-18] +* AL  *unc-64;atg-18;Ex[Pges-1::atg-18] +* DR  *unc-31 +* AL  *unc-31 +* DR | 24,18  27,23  25,23  30,25  15,18  15,18  25,24  30,29  22,21  28,23  28,20  28,27 | 34,27  41,33  37,33  41,34  22,23,  22,24  38,36  51,38  32,29  34,32  39,32  43,33 | /  113%, 128%  /  120%,109%  /  100%,100%  /  120%,121%  /  127%,110%  /  100%,135% | 118(3),78(11)  100(30),95(11)  111(7),91(24)  117(8),102(8)  88(11),84(15)  95(9),104(10)  135(14),118(3)  135(3),92(16)  125(16),93(13)  68(22),89(13)  130(2),69(28)  92(11),62(15) | /  <0.0001,<0.0001  /  <0.0001,0.0006  /  0.075,0.6679  /  <0.0001,<0.0001  /  0.0013,0.0012  /  0.0219, <0.0001 |
| *unc-31;atg-18+* AL  *unc-31;atg-18+* DR  *unc-31;atg-18;Ex[Punc-119::atg-18] +* AL  *unc-31;atg-18;Ex[Punc-119::atg-18] +* DR  *unc-31;atg-18;Ex[Pges-1::atg-18] +* AL  *unc-31;atg-18;Ex[Pges-1::atg-18]*+ DR | 22,18  24,18  28, 18  25,25  23,20  24,20 | 34,25  34,27  39,30  46,32  36,27  33,29 | /  109%,100%  /  89%,139%  /  104%,100% | 105(10),87(8)  87(8),88(7)  121(10),67(7)  113(13),65(8)  122(16),84(9)  111(19),84(11) | /  0.3222,0.276  /  0.0387, <0.0001  /  0.2013,0.2416 |

*^a^* Median lifespan for each trial

*^b^* Maximum lifespan for each trial

*^c^* Percentage of changes in median lifespan (DR) relative to corresponding control (AL) for each trial

*^d^* Numbers of animals counted for each trial (censored: animals died of internal hatching or lost during the experiments)

*^e^* *p* values (log-rank test) compared to corresponding control (DR vs. AL)
