# Supplementary material for: The cell non-autonomous function of ATG-18 is essential for neuroendocrine regulation of Caenorhabditis elegans lifespan
Source: PLoS Genet. 2017 May 30;13(5):e1006764. doi: 10.1371/journal.pgen.1006764 (PMC5469504; doi:10.1371/journal.pgen.1006764)
Supplement: S12 Table — (DOCX) [file pgen.1006764.s022.docx]

**S12 Table. Statistical analysis of lifespan data for Fig 8A**

| **Genotype** | **RNAi** | **Lifespan (days)** | | **% of control *^c^*** | **n *^d^***  **(censored)** | ***p* *^e^*** |
| --- | --- | --- | --- | --- | --- | --- |
|  |  | **median *^a^*** | **max *^b^*** |  |  |  |
| *daf-2* | vector | 28,37 | 48,49 | / | 85(3),79(10) | / |
| *daf-2* | *daf-16* | 14,14 | 16,16 | 50%,38% | 72(17),75(8) | <0.0001,<0.0001 |
| *daf-2;atg-18* | vector | 18,20 | 32,28 | / | 74(8),75(8) | / |
| *daf-2;atg-18* | *daf-16* | 11,11 | 16,16 | 61%,55% | 75(10),80(0) | <0.0001,<0.0001 |
| *daf-2;atg-18;Ex[Punc-119::atg-18]*  *daf-2;atg-18;Ex[Punc-119::atg-18]* | *vector*  *daf-16* | 28,25  14,16 | 49,49  21,21 | /  50%,64% | 78(2),67(10)  72(6),69(1) | /  <0.0001,<0.0001 |
| *daf-2;atg-18;Ex[Pges-1::atg-18]*  *daf-2;atg-18;Ex[Pges-1::atg-18]* | *vector*  *daf-16* | 29,29  14,16 | 49,45  21,21 | /  48%,55% | 80(9),87(0)  78(1),89(2) | /  <0.0001,<0.0001 |

*^a^* Median lifespan for each trial

*^b^* Maximum lifespan for each trial

*^c^* Percentage of changes in median lifespan relative to corresponding vector control for each trial

*^d^* Numbers of animals counted for each trial (censored: animals died of internal hatching or lost during the experiments)

*^e^* *p* values (log-rank test) compared to corresponding vector control
