# Supplementary material for: The cell non-autonomous function of ATG-18 is essential for neuroendocrine regulation of Caenorhabditis elegans lifespan
Source: PLoS Genet. 2017 May 30;13(5):e1006764. doi: 10.1371/journal.pgen.1006764 (PMC5469504; doi:10.1371/journal.pgen.1006764)
Supplement: S13 Table — (DOCX) [file pgen.1006764.s023.docx]

**S13 Table. Influence of *atg-18* mutations on the expression levels of ILP genes**

| **ILP genes** | ***daf-2* v.s.**  ***daf-2;atg-18^a^*** | ***daf-2;atg-18* v.s.**  ***daf-2;atg-18;Ex[Punc-119::atg-18]^a^*** | ***daf-2;atg-18* v.s.**  ***daf-2;atg-18;Ex[Pges-1::atg-18]^a^*** |
| --- | --- | --- | --- |
| ***ins-1*** | **0.0073, 0.0134** | 0.1038, 0.2315 | **0.0232, 0.0034** |
| *ins-2* | 0.3498, 0.1833 | / | / |
| ***ins-3*** | **0.0141, 0.0194** | 0.0548, 0.7413 | 0.6673, 0.7220 |
| *ins-4* | 0.0827, 0.3758 | / | / |
| *ins-5* | 0.0726, 0.5656 | / | / |
| *ins-6* | 0.0798, 0.5634 | / | / |
| *ins-7* | 0.0647, 0.8617 | / | / |
| *ins-8* | 0.2468, 0.2439 | / | / |
| *ins-9* | 0.0747, 0.7048 | / | / |
| *ins-10* | 0.2761, 0.9621 | / | / |
| *ins-11* | 0.3621, 0.8275 | / | / |
| *ins-12* | 0.9373, 0.9628 | / | / |
| *ins-13* | 0.2675, 0.3438 | / | / |
| *ins-14* | 0.2064, 0.2221 | / | / |
| *ins-15* | 0.5669, 0.7389 | / | / |
| *ins-16* | 0.2508, 0.5255 | / | / |
| *ins-17* | 0.3272, 0.3849 | / | / |
| *ins-18* | 0.2579, 0.1927 | / | / |
| *ins-19* | 0.2504, 0.0524 | / | / |
| *ins-20* | 0.2657, 0.1964 | / | / |
| *ins-21* | 0.529, 0.2085 | / | / |
| *ins-22* | 0.4458, 0.5791 | / | / |
| *ins-23* | 0.6356, 0.3714 | / | / |
| *ins-24* | 0.1108, 0.2291 | / | / |
| *ins-25* | 0.2086, 0.3990 | / | / |
| *ins-26* | 0.5679, 0.3615 | / | / |
| *ins-27* | 0.2834,0.4945 | / | / |
| *ins-28* | 0.7437, 0.1412 | / | / |
| ***ins-29*** | **0.0062, 0.0036** | **0.0044, 0.0177** | **0.001, 0.0125** |
| *ins-30* | 0.3788, 0.6082 | / | / |
| *ins-31* | 0.2202, 0.1133 | / | / |
| *ins-32* | 0.5303, 0.3001 | / | / |
| *ins-33* | 0.2284, 0.4704 | / | / |
| *ins-34* | 0.2639, 0.1272 | / |  |
| ***ins-35*** | **0.0034, 0.0030** | **0.0161, 0.0379** | 0.1052,0.3488 |
| *ins-36* | 0.5126, 0.2603 | / | / |
| ***ins-37*** | **0.0036, 0.0001** | 0.3611,0.6729 | **0.0344, 0.0090** |
| *ins-38* | 0.1712, 0.2227 | / | / |
| *ins-39* | 0.1638, 0.8364 | / | / |
| ***daf-28*** | **0.0074, 0.0037** | **0.0070, 0.0201** | **0.0004, 0.0009** |

*^a^* *p* values (t-test) compared to corresponding control
